# Synthesis and insecticidal activity of some pyrazole, pyridine, and pyrimidine candidates against *Culex pipiens* L. larvae

**DOI:** 10.1038/s41598-025-27807-y

**Published:** 2025-11-29

**Authors:** Eman A. E. El-Helw, Eman A. El-Bordany, Doaa R. Abdel-Haleem, Sara F. El-Fagal, Eman A. Ghareeb

**Affiliations:** 1 Chemistry Department, Faculty of Science, Ain Shams University, Cairo, 11566 Egypt; 2 Entomology Department, Faculty of Science, Ain Shams University, Cairo, 11566 Egypt

**Keywords:** Benzo[*f*]quinolines, Pyrazolines, Culex pipiens, Insecticidal activity, Resistance level, Biochemistry, Chemical biology, Chemistry, Drug discovery

## Abstract

The expansion of the *Culex pipiens* population has increased the spread of arboviruses, due to the ineffectiveness of several traditional insecticides available to control this vector. Some heterocycles bearing a benzoquinoline moiety, such as pyrazoline, pyridine, pyridone, and pyrimidine derivatives, were synthesized from the reaction of 3-(3-chlorobenzo[f]quinolin-2-yl)-1-(4-chlorophenyl)prop-2-en-1-one with some nitrogen and carbon nucleophiles. The synthesized compounds were investigated against laboratory and field strains of *C. pipiens* larvae. The activities of prepared derivatives were varied, where compounds **7**, **5**, and **9** showed good toxicities with LC_50_ of 180.35, 222.87, and 235.25 µg/mL, respectively, for the lab strain. At the same time, their potencies decreased to 242.44, 265.83, and 280.84 µg/mL, respectively, for the field strain. In contrast, pyridone **10** was the least effective compound with an LC_50_ of 336.93 and 346.60 µg/mL for the lab and field strain, respectively. The field strain exhibited low resistance levels against all tested compounds. Structure-activity relationship was studied to demonstrate the effect of each substitution on activity against *C. pipiens.* Therefore, more substitutions should be inserted to enhance the activity of chalcone to be used in the mosquito larvae control programs, especially the field strain showed low resistance levels.

**Supplementary Information:**

The online version contains supplementary material available at 10.1038/s41598-025-27807-y.

## Introduction

Mosquitoes are considered the most dangerous animals to humans due to the high mortality and morbidity they cause in both humans and animals. While their aquatic immature stages serve as a food source for aquatic organisms, the severe impact of mosquitoes on public health cannot be overlooked^1^. *Culex pipiens* L. (Diptera: Culicidae) is the most prevalent mosquito species in the Northern Hemisphere. Its presence is strongly influenced by water habitats rich in organic matter^2^. *Culex pipiens* shows high adaptability to various environments, leading to its global distribution as a disease vector^3^. This species plays a key role in transmitting several pathogens, including viruses such as St. Louis encephalitis and West Nile virus, filarial worms, and avian malaria (Plasmodium spp.)^4^.

Selecting proper insecticides is one of the key requirements for controlling public health pests, which ensures more specificity to key pests and less toxicity to humans and non-targets. Recently, most larvicides used to control mosquito larvae are toxic. So, many efforts are exerted to achieve an effective, eco-friendly, and low-cost active ingredient^5^.

Quinolines and their benzo-fused candidates are found in many natural products and pharmaceuticals. They are significant due to their chemical and biological properties^6–15^, including insecticidal effects^16–20^. These compounds are often used in the synthesis of pharmaceuticals, dyes, and ligands in different fields. They exhibit a range of biological activities depending on their substitution pattern. On the other hand, chalcone (α,β-unsaturated carbonyl system) is an organic compound belonging to the flavonoid family and serves as a key intermediate in the biosynthesis of many natural products and pharmaceuticals.

Chalcones are notable for their simplicity and versatility, and they form the backbone for various biologically active molecules such as insecticidal, anticancer, anti-inflammatory, antioxidant, antimicrobial, and antimalarial agents^21–27^. The compounds containing hydrazinyl, thioxoacetamide, phenyl chloride showed good activity against *Spodoptera littoralis* larvae and had binding affinity to the target enzyme^28^. In addition the pyridine derivatives had aphicidal activities against nymphs and adults of the cotton aphid^29^. Therefore, this work aimed to synthesize chalcone block bearing a benzoquinoline core to investigate its activity towards nucleophilic reagents and the insecticidal activity against lab and field strains of *C. pipiens* larvae, in addition to, detection of resistance levels in the field strain.

## Results and discussion

### Chemistry

**Scheme 1:**
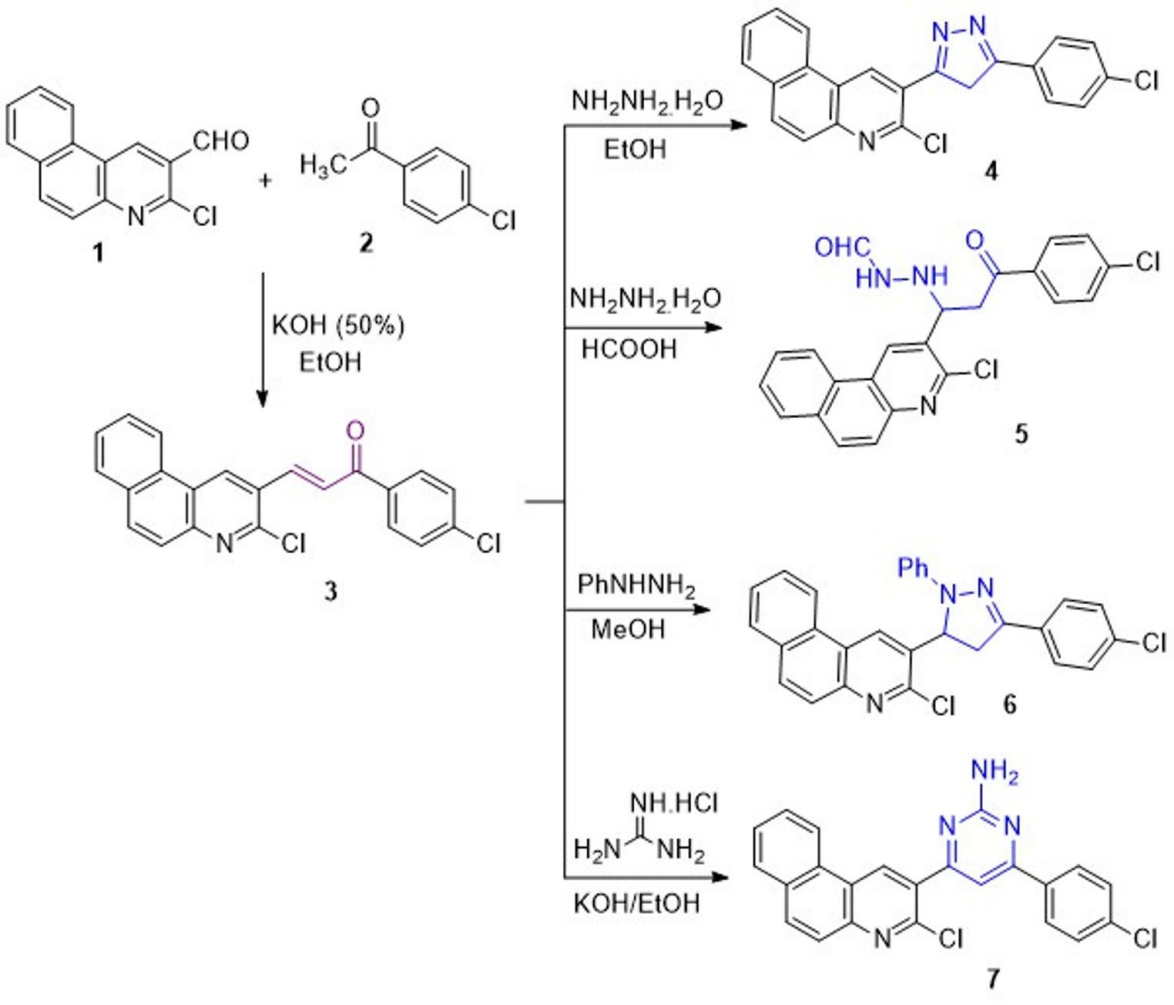
Synthesis of chalcone **3** and its reactions with some nitrogen nucleophiles.

The chalcone derivative **3** was provided by base-catalyzed Claisen-Schmidt condensation reaction of benzo[*f*]quinoline-2-carbaldehyde **1**^30^ with *p*-chloroacetophenone **2** in the presence of alcoholic potassium hydroxide^25,31^ (Scheme 1). The chalcone derivative **3** was subjected to react with some nitrogen nucleophilic reagents to synthesize new heterocyclic derivatives with evaluation of their insecticidal activity. Initially, hydrazinolysis of chalcone **3** with hydrazine hydrate depends on the reaction conditions (Scheme 1). Carrying out the reaction in refluxing ethanol afforded the pyrazole derivative **4** *via* aza Michael’s addition of hydrazine-amino group on β-carbon of unsaturated carbonyl group of chalcone **3**, followed by *exo*-trig cyclization to remove water molecule and then dehydrogenation. When the same reaction was carried out in refluxing formic acid^31^, the *N*-formylhydrazine derivative **5** was produced.

**Scheme 2:**
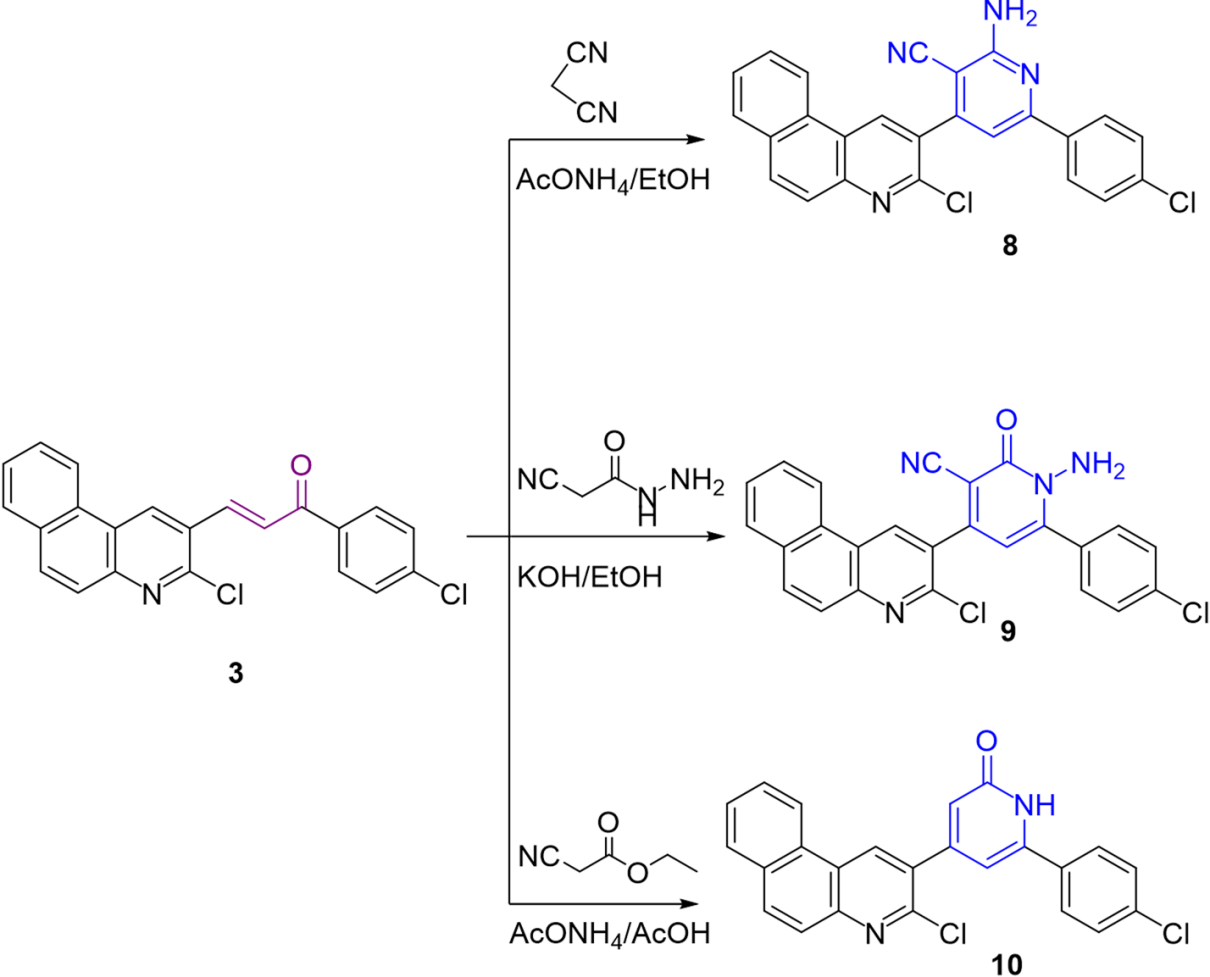
Reactions of chalcone derivative **3** with some carbon nucleophiles.

The IR spectrum of pyrazole **4** lacked the absorption band for the carbonyl group. Its ^1^H-NMR spectrum exposed a singlet signal at δ 3.09 ppm for methylene protons, which supported the formation of a pyrazole ring. The IR spectrum of compound **5** exhibited two absorption bands at υ 1692 and 1663 cm^− 1^ for carbonyl groups, and its ^1^H-NMR revealed two exchangeable singlet signals for two NH protons at δ 9.30 and 8.78 ppm, in addition to another singlet signal at δ 8.62 ppm for CHO proton.

On the other hand, heating chalcone **3** with phenylhydrazine in methanol furnishes *N*-phenylpyrazole derivative **6** (Scheme 1). Its structure was confirmed from its spectral data. In turn, the 2-aminopyrimidine **7** was established from the treatment of chalcone **3** with guanidine hydrochloride in ethanol in the presence of potassium hydroxide. The ^1^H-NMR spectrum of compound **7** showed an exchangeable singlet signal for the primary amino group at δ 6.85 ppm.

The behavior of chalcone **3** towards some carbon nucleophiles, such as malononitrile, 2-cyanoacetohydrazide, and ethyl cyanoacetate, was studied. Thus, the enamino nitrile derivative **8** was achieved by treating chalcone **3** with malononitrile in ammonium acetate and ethanolic solution. Heating chalcone **3** with 2-cyanoacetohydrazide in alcoholic potassium hydroxide gave the cyanopyridone **9** (Scheme 2). The IR spectrum of compound **9** demonstrated two bands at υ 2201 & 1710 cm^− 1^ for cyano and carbonyl groups, respectively. Its ^1^H-NMR spectrum exposed two singlet signals for CH pyrimidine and NH_2_ at δ 8.70 & 7.07 ppm. The pyridine-2-one **10** was furnished upon treating chalcone **3** with ethyl cyanoacetate in refluxing acetic acid involving ammonium acetate *via* Michael addition followed by cyclization.

### Insecticidal activity

The toxicity of prepared derivatives was screened against lab and field strains of *C. pipiens* larvae. Compounds **7**,** 5**, and **9** were more potent, with LC_50_ values of 180.35, 222.87, and 235.25 µg/mL, respectively, for the lab strain. At the same time, the activity decreased to 242.44, 265.83, and 280.84 µg/mL, respectively, for the field strain after 48 h (Tables 1 and 2). Chalcone **3** showed moderate mortality with LC_50_ 258.49 and 289.28 µg/mL against lab and field strains, respectively, 48 h post-treatment. Some moieties markedly affected biological activity, where the larvicidal potency of **8**,** 6**,** 4**, and **10** was reduced compared to chalcone **3**.

**Fig. 1:**
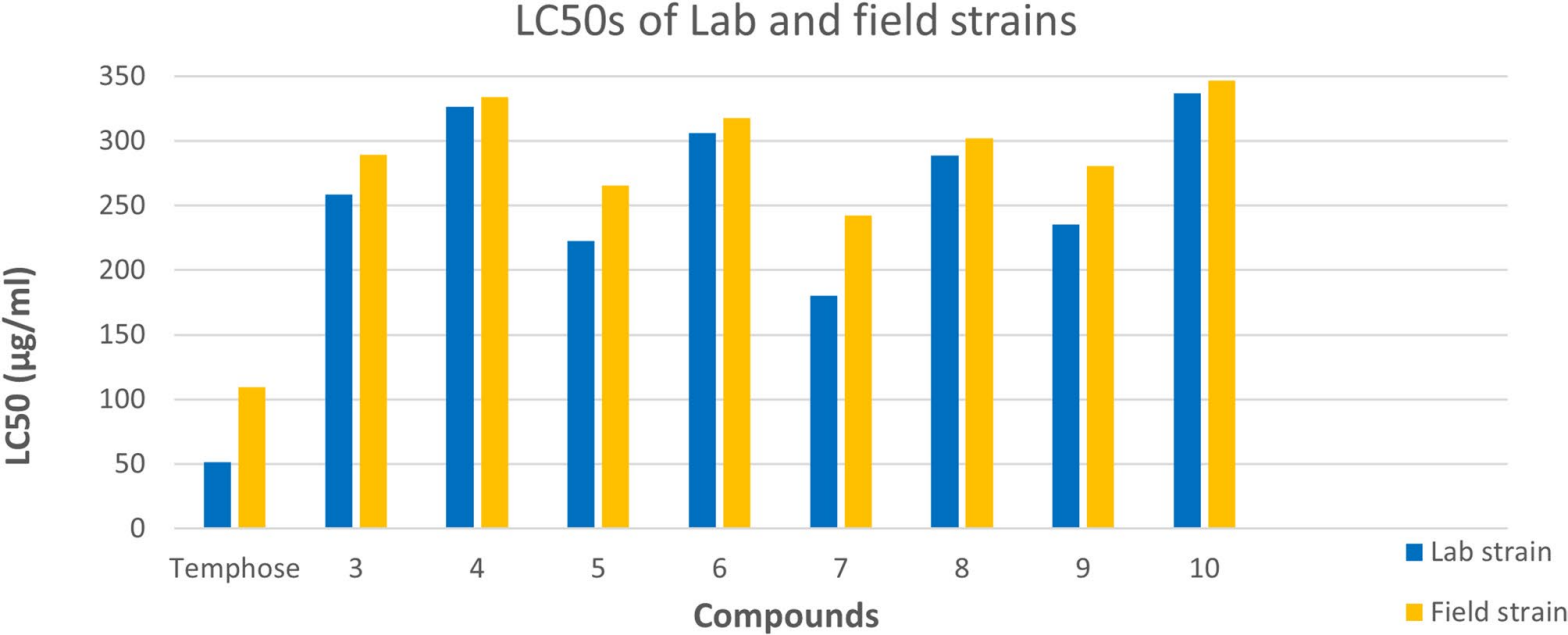
LC_50_ values for lab and field strains of *C. pipiens* larvae after treatment with prepared derivatives 48 h post-treatment.

The LC_50_ values of **8**,** 6**,** 4**, and **10** were 288.96, 306.34, 326.52, and 336.93 µg/mL, respectively, for the lab strain and 302.26, 317.75, 333.91, and 346.60 µg/mL for the field strain. The variations in LC_50_ values between lab and field strains were demonstrated in Fig. 1. The resistance ratios of the field strain were low for all tested compounds because their values were less than **5** (Table 2). The toxicity of temephos for the field strain was 2.12 times that of the lab strain, which was higher than all the tested compounds. Both strains were homogenous in their response to all tested compounds except temphose against the lab strain.

**Table 1:** Toxicity of the prepared derivatives against the lab strain of the third larval instar of *Culex pipiens* after 48 h of exposure.

Compd. (µg/mL)	LC_25_ (^a^F.l. at 95%)	LC_50_ (^a^F.l. at 95%)	LC_90_ (^a^F.l. at 95%)	^b^Slope ± SE	^c^X^2^	*P*
**3**	157.72 (118.30- 180.21)	258.49 (219.48- 311.94)	660.89 (571.74- 1099.18)	3.14 ± 0.28	14.17	0.02
**4**	205.08 (186.85–221.40)	326.52 (302.32- 358.71)	790.17 (654.94- 1033.34)	3.33 ± 0.32	1.06	0.95
**5**	141.58 (91.80- 158.73)	222.87 (173.46- 277.71)	527.79 (492.64- 948.26)	3.42 ± 0.28	6.64	0.01
**6**	184.10 (164.95- 200.78)	306.34 (282.92–336.80)	806.16 (660.20- 1074.87)	3.04 ± 0.30	4.26	0.51
**7**	119.84 (105.78- 132.15)	180.35 (167.38- 192.67)	392.07 (355.85- 443.65)	3.80 ± 0.28	9.71	0.08
**8**	169.17 (149.47- 186.06)	288.96 (266.62- 317.15)	799.17 (651.67- 1073.43)	2.90 ± 0.29	4.19	0.52
**9**	148.76 (94.95- 166.96)	235.25 (183.24- 300.08)	561.95 (536.82- 1088.14)	3.38±0.29	8.09	0.01
**10**	214.08 (195.97–230.50)	336.93 (311.78- 370.91)	797.59 (661.16- 1043.60)	3.42 ± 0.33	0.97	0.96
**Temephos**	27.64 (14.32–32.99)	51.58 (34.84–64.85)	168.75 (140.97–313.50)	2.48 ± 0.22	12.99	0.01

^a^ (F.l.) Fiducial limits.

^b^Slope of the concentration-mortality regression line ± standard error.

^c^*X*^*2*^ chi-square significant at *P* < 0.05.

**Table 2:** Toxicity of the prepared derivatives against the field strain of the third larval instar of *Culex pipiens* after 48 h of exposure.

Compd. (µg/mL)	LC_25_ (^a^F.l. at 95%)	LC_50_ (^a^F.l. at 95%)	LC_90_ (^a^F.l. at 95%)	^b^Slope ± SE	^c^X^2^	*P*	^d^RR
**3**	203.03 (185.33–217.80)	289.28 (273.34- 307.48)	566.86 (499.63- 677.77)	4.38 ± 0.42	1.38	0.84	1.11
**4**	237.85 (220.89- 252.63)	333.91 (314.65- 358.82)	636.14 (552.88- 779.08)	4.57 ± 0.45	0.92	0.92	1.02
**5**	184.98 (166.70- 200.02)	265.83 (250.68- 281.88)	529.48 (469.66- 626.78)	4.28 ± 0.40	3.83	0.42	1.19
**6**	223.73 (206.28–238.60)	317.75 (299.67- 340.26)	618.83 (538.87- 755.12)	4.42 ± 0.43	0.47	0.97	1.03
**7**	167.00 (147.99- 182.52)	242.44 (227.33- 257.26)	492.25 (439.01- 578.06)	4.16 ± 0.39	5.86	0.21	1.34
**8**	212.82 (195.33- 227.55)	302.26 (285.54- 322.11)	588.67 (516.54- 709.07)	4.42 ± 0.42	0.89	0.92	1.04
**9**	193.24 (174.48- 208.67)	280.84 (264.65- 298.98)	571.45 (500.47- 690.94)	4.15 ± 0.41	2.48	0.64	1.19
**10**	251.21 (234.87- 265.81)	346.60 (326.70- 372.91)	638.92 (556.82- 779.36)	4.82 ± 0.47	1.02	0.91	1.03
**Temephos**	65.25 (57.81–71.89)	109.39 (100.15- 121.11)	292.02 (238.92- 388.55)	3.00 ± 0.27	7.03	0.13	2.12

^a^ (F.l.) Fiducial limits.

^b^Slope of the concentration-mortality regression line ± standard error.

^c^*X*^*2*^ chi-square significant at *P* < 0.05.

^d^(RR) resistance ratio.

Chalcone is one of the most abundant natural products found in many plant species. It belongs to the flavonoids, which are well-known functional secondary metabolites with insect antifeedant activity^32^. It is a bioactive compound used for the synthesis of various biologically active molecules that are reported to have many pharmacological applications, larvicidal, and insect antifeedant activities^5^. They were investigated for antifeedant, and toxicity impacts on *Spodoptera frugiperda* larvae^33^, larvicidal activity, and juvenile hormone antagonists against *Aedes albopictus*^34^.

4-Aminoacetophenone-chalcone derivatives were effective against *Anastrepha suspensa* females, where cinnamoyl phenyl urea showed great insecticidal toxicity (73% mortality at 100 µg/female after 24 h), while other derivatives exhibited less than 30% mortality^35^. In addition, some chalcones were moderately to highly repellent against *Callosobruchus maculatus*^36^. All tested chalcone derivatives had LC_50_ values less than 100 mg /mL at 24 h post-treatment, which is the concentration the WHO recommends for selecting promising larvicides. Some substituted chalcones were effective larvicides against *Ae. Aedes aegypti*, with no toxicity to non-target animal models^37^. Moreover, the presence of a chlorine atom attached to the aromatic cycle may be the key reason for the high effectiveness of the compounds against the larval instars of *S. littoralis*^38^. Pyridines and pyrimidines derivatives showed high activities against *Aphis gossypii* nymphs and adults, with a remarkable effect on the histology of aphids’ digestive system^39,40^. Also, the hydrazide-bearing compounds exhibited toxicological effects against the second and fourth larval instars of *Spodoptera littoralis*. Besides they had adverse impacts on activity of many enzymes involved in the cuticle synthesis of *S. littoralis* larvae^41^.

### Structure-activity relationship

Chalcone derivative has a skeletal framework for synthesizing many bioactive heterocyclic structures through ring closure and condensation reactions^5^. The larvicidal potency of the chalcone derivatives depended on the type, number, physicochemical properties, and position of substituents on the enone group^37^. The electrophilic nature of the unsaturated carbonyl group in chalcone is the key factor in forming irreversible bonds with other moieties, resulting in different bioactive structures. This reactivity could be affected by the insertion of an X-substituent in the enone group or by moieties inserted on aromatic rings^42^. Chalcones exhibited high reactivity due to the enone system, where most substituents were inserted.

The insertion of different moieties into the chalcone derivative **3** affected its toxicity; compounds **5**,** 7**, and **9** had good larvicidal activity, while **8**,** 6**, and **10** showed moderate to low insecticidal potencies at 48 h post-treatment. The formation of 2-aminopyrimidine in the derivative **7** increases its activity, the attached amino group donates electrons and might help in resonances in the pyrimidine ring^43^. The electron donor group (-OCH_3_) linked to the ring was responsible for the larvicidal potency against *Ae. Aedes aegypti* larvae^37^.

The formation of formyl hydrazide in **5** improves its activity; the presence of electron-withdrawing formyl and ketone groups and 2 electron-donating amino groups increases the positivity and reactivity with detoxifying enzymes. Cyanopyridone derivative **9** contains 3 functional groups: cyano, carbonyl (electron-withdrawing groups), and amino (electron-donating group) groups, which elevate resonance on pyridine and might be responsible for its toxicity^44^. On the other hand, the absence of carbonyl in the derivative **8** decreased its activity than **9** and greatly declined in **10** due to the absence of both amino and cyano groups. The presence of pyrazole, in addition to the bulking of the *N*-phenyl pyrazole moiety in derivative **6**, causes its low potency^45^. In conclusion, the biological activity of chalcone derivatives was mainly enhanced by the presence and position of electron-donating groups (e.g., amino, -OCH_3_) that increase resonance in the rings, therefore the reactivity. Also, the electron-withdrawing groups as formyl, carbonyl and cyano, elevated molecular positivity and interaction with target enzymes. On the other hand, the absence of key groups like carbonyl reduces activity, so it is necessary to maintain the enone system. In addition, the formation of specific heterocyclic rings, like 2-aminopyrimidine, boosted the activity, while bulky substituents like pyrazole moieties tend to lower potency^46^.

## Conclusion

A series of benzoquinoline-based heterocycles like pyrazoles, pyrimidines, and pyridines was synthesized through cyclocondensation reactions of chalcone with some nitrogen and carbon nucleophiles. The toxicity of prepared derivatives was screened against lab and field strains of *C. pipiens* larvae. The compounds **7**,** 5**, and **9** were more potent for the lab strain. At the same time, the activity decreased for the field strain after 48 h. Chalcone **3** had moderate mortality with LC_50_ 258.49 and 289.28 µg/mL against lab and field strains, respectively. SAR study revealed that electron-donating and withdrawing moieties markedly affected biological activity, where the larvicidal potency of certain compounds was reduced compared to chalcone **3**. The toxicity of temphose for the field strain was 2.12 times that of lab strain, which was higher than all the tested compounds. Both strains were homogenous in their response to all tested compounds except temphose against the lab strain. Continuing efforts are focused on further considering their biological activities and improving their potency through structural optimization.

## Materials and methods

### Chemistry

Purification and drying of solvents and chemicals, obtained from Sigma Aldrich, Merck, Fluka, and El-Nasr pharmaceutical chemicals companies, were performed by standard techniques. The GALLENKAMP electric melting point apparatus was used to measure the melting points of synthesized compounds. The infrared spectra were performed using KBr disks on FTIR Thermo Electron Nicolet iS10 (USA) infrared spectrometer and expressed in wavenumber (*ν*, cm^− 1^) at the Faculty of Science, Ain Shams University. The ^1^H and ^13^C NMR spectra were recorded at 400 and 100 *MHz* on a BRUKER NMR spectrometer using tetramethyl silane (TMS) as an internal standard in deuterated dimethyl sulfoxide (DMSO-*d*_*6*_) at the Faculty of Pharmacy, Ain Shams University. Chemical shifts (δ) are quoted in ppm. Mass spectra were performed on a Shimadzu GC-MS-QP-1000 EX mass spectrometer operating at 70 eV at the Faculty of Science, Cairo University, Egypt. Elemental analyses were measured at the microanalytical unit, Faculty of Science, Ain Shams University. The reactions were monitored by thin-layer chromatography (TLC) using Merck Kiesel gel 60 F_254_ obtained from Fluka, Switzerland.

### (E)-3-(3-Chlorobenzo[f]quinolin-2-yl)-1-(4-chlorophenyl)prop-2-en-1-one (3)

To a mixture of 4-chloroacetophenone **2** (0.01 mol, 1.54 mL) and KOH (50%) in methanol, the aldehyde **1** (0.01 mol, 2.41 g) was added portion-wise, and then the reaction mixture was stirred at room temperature for 8 h. The solid obtained was filtered, washed with water, dried, and recrystallized from dioxane to afford beige crystals, mp. 182–184 °C, Yield (90%), Anal. Calcd. for C_22_H_13_Cl_2_NO (378.3): C, 69.86; H, 3.46; N, 3.70. Found: C, 69.90; H, 3.42; N, 3.73%. FTIR (ν, cm^− 1^): 1662 (C = O). ^1^H NMR (DMSO-*d*_6_, *δ*, ppm): 9.30 (d, 1H, C5-H, J = 7.2 *Hz*), 9.01 (s, 1H, C4-H quinoline), 8.25–7.83 (m, 9H, Ar-H), 7.92 (d, 1H, CHβ, *J* = 7.0 *Hz*), 7.70 (d, 1H, CHα, *J* = 7.0 *Hz*). ^13^C-NMR (DMSO-*d*_6_, *δ*, ppm): 197.65 (C = O), 150.11 (C = N), 143.98 (C_β_=C), 138.76 (C-Cl), 137.29 (C = C_α_), 135.55, 133.71, 130.42 (2), 129.91 (2), 129.29 (2), 129.14, 128.59, 128.55 (2), 127.98 (2), 125.91, 125.19, 123.98 (Ar-C).The mass(*m/z%*):378 (26.81), 346(19.11), 264(50.03), 130(62.32), 101.93(100).

### 3-Chloro-2-(5-(4-chlorophenyl)-4H-pyrazol-3-yl)benzo[f]quinoline (4)

A solution of chalcone **3** (0.01 mol, 3.78 g) and hydrazine hydrate in ethanol (20 mL) was refluxed for 8 h. The solid obtained was filtered and recrystallized from ethanol to furnish beige crystals, mp. 254–256 °C, Yield (88%), Anal. Calcd. for C_22_H_13_Cl_2_N_3_ (390.3): C, 67.71; H, 3.36; N, 10.77. Found: C, 67.74; H, 3.39; N, 10.74%. FTIR (ν, cm^− 1^): 1620 (C = N). ^1^H NMR (DMSO-*d*_6_, *δ*, ppm): 9.02 (s, 1H, C4-H quinoline), 8.44 (d, 1H, C5-H), 8.05–7.43 (m, 9 H, Ar-H), 3.09 (s, 2 H, CH_2_).The mass(*m/z%*):390(21.72), 376.16(83.38), 227.25(85.55), 202.19(100), 160.39(69.11), 110.75(74.71).

### N’-(1-(3-Chlorobenzo[f]quinolin-2-yl)-3-(4-chlorophenyl)-3-oxopropyl)formo-hydrazide (5)

A mixture of chalcone **3** (0.01 mol, 3.78 g) and hydrazine hydrate in formic acid (20 mL) was heated under reflux for 12 h. The formed precipitate was filtered and recrystallized from ethanol to produce yellow crystals, mp. 172–174 °C, Yield (75%), Anal. Calcd. for C_23_H_17_Cl_2_N_3_O_2_ (338.3):C, 63.03; H, 3.91; N, 9.59. Found: C, 63.05; H, 3.89; N, 9.61%. FTIR (ν, cm^− 1^): 3105 (NH), 1692 (C = O ketone), 1663 (C = O amide). ^1^H NMR (DMSO-*d*_6_, *δ*, ppm): 9.30 (s, 1H, *NH*CO, exchangeable), 8.99 (s, 1H, C4-H quinoline), 8.78 (s, 1H, NH, exchangeable), 8.62 (s, 1H, CHO), 8.26–7.52 (m, 10 H, Ar-H), 4.46 (t, 1H, CH, J= 7.00 *Hz*), 3.68 (d, 2 H, CH_2_, J= 7.00 Hz). ^13^C-NMR (DMSO-*d*_6_, *δ*, ppm): 197.65 (C = O), 150.11 (C = O formyl), 143.97 (C = N), 138.76 (C-Cl), 137.29, 135.55, 133.70, 130.42, 129.91, 129.28 (2), 129.14, 128.59 (2), 128.54, 127.98 (2), 125.91 (2), 125.18 (2), 123.98 (2) (Ar-C).The mass(*m/z%*):338.01(73.99), 313.08(93.59), 256.35(96.30), 209.29(42.35), 157.96(60.37), 100.82(48.41), 48.62(100).

### 3-chloro-2-(3-(4-chlorophenyl)-1-phenyl-4,5-dihydro-1 H-pyrazol-5-yl)benzo[f]quinoline (6)

A mixture of chalcone **3** (0.01 mol, 3.78 g) and phenylhydrazine (0.01 mol, 1.08 mL) in methanol (20 mL) was heated under reflux for 8 h. The formed precipitate was filtered and recrystallized from ethanol to afford pale-yellow crystals, 208–210 °C, Yield (85%). Anal. Calcd. for C_28_H_19_Cl_2_N_3_ (468.4): C, 71.80; H, 4.09; N, 8.97. Found: C, 71.78; H, 4.11; N, 8.95%. FTIR (ν, cm^− 1^): 1624 (C = N). ^1^H NMR (DMSO-*d*_6_, *δ*, ppm): 9.02 (s, 1H, C4-H quinoline), 8.14–6.77 (m, 15 H, Ar-H), 5.86–5.80 (dd, 1H, CH pyrazole, *J* = 6.50 and 6.90 *Hz*), 4.19–4.09 (dd, 1H, CH_a_, *J* = 6.50 and 10 *Hz*), 3.39–3.33 (dd, 1H, CH_b_, *J* = 6.90 and 10 *Hz*).The mass(*m/z%*):468.07(33.62),348.07(25.25), 264.41(22.28),109.26(75.46), 88.25(100).

### 4-(3-chlorobenzo[f]quinolin-2-yl)-6-(4-chlorophenyl)pyrimidin-2-amine (7)

Guanidine hydrochloride (0.01 mol, 0.95 g) was added to a solution of chalcone **3** (0.01 mol, 3.78 g) in alcoholic KOH (10%), and then the reaction mixture was heated under reflux for 10 h. The solution was poured into cold water, and the solid obtained was filtered, washed with water, dried, and recrystallized from ethanol to give yellow crystals, mp. 225–227 °C, and Yield (80%). Anal. Calcd. for C_23_H_14_Cl_2_N_4_ (416.1): C, 66.22; H, 3.38; N, 13.43. Found: C, 66.24; H, 3.35; N, 13.45%. FTIR (ν, cm^− 1^): 3317, 3192 (NH_2_), 1625 (C = N). ^1^H NMR (DMSO-*d*_6_, *δ*, ppm): 9.08 (d, 1H, C5-H, *J* = 7.0 *Hz*), 8.89 (s, 1H, C4-H quinoline), 8.14–7.60 (m, 10 H, Ar-H + CH pyrimidine), 6.85 (s, 2 H, NH_2_, exchangeable). ^13^C NMR (DMSO-*d*_6_, *δ*, ppm): 163.97 (C = N), 162.99 (C = N), 162.36 (C = N), 158.85 (C-Cl), 144.05, 139.46, 136.20, 135.29, 133.98, 129.58 (2), 128.93, 128.48 (2), 127.97, 126.76 (2), 125.51, 125.16, 124.17, 121.72, 121.59, 106.04 (Ar-C).).The mass(*m/z%*):415.59(17.98), 393.25(39.80), 284.64(45.83), 237.70(100), 181.62(79.81), 99.54(66.36).

### 2-Amino-4-(3-chlorobenzo[f]quinolin-2-yl)-6-(4-chlorophenyl)nicotinonitrile (8)

A mixture of chalcone **3** (0.01 mol, 3.78 g), malononitrile (0.01 mol, 0.66 g), and ammonium acetate (0.03 mol, 2.31 g) in ethanol (25 mL) was heated under reflux for 10 h. The formed precipitate was filtered and recrystallized from ethanol to produce beige precipitate, mp.176–178 °C °C, Yield (85%). Anal. Calcd. for C_25_H_14_Cl_2_N_4_ (441.3):C, 68.04; H, 3.20; N,12.70. Found: C, 68.06; H, 3.18; N, 12.74%. FTIR (ν, cm^− 1^): 3397, 3328 (NH_2_), 2222 (C ≡ N), 1620 (C = N). ^1^H NMR (DMSO-*d*_6_, *δ*, ppm): 9.01 (d, 1H, C5-H quinoline, *J* = 7.1 *Hz*), 8.77 (s, 1H, C4-H quinoline), 8.34–7.35 (m,10 H, Ar-H + CH pyrimidine), 5.00 (s, 2 H, NH_2,_ exchangeable).The mass(*m/z%*): 441.62(31.49), 377.86(72.98), 327.43(100), 251.10(68.36), 147(61.15), 98.98(99.87).

### 1-Amino-4-(3-chlorobenzo[f]quinolin-2-yl)-6-(4-chlorophenyl)-2-oxo-1,2-dihydropyridine-3-carbonitrile (9)

A mixture of chalcone **3** (0.01 mol, 3.78 g), 2-cyanoacetohydrazide (0.01 mol, 0.99 g) in alcoholic KOH (10%) was heated under reflux for 10 h. After cooling, the reaction mixture was poured into ice-cold water. The deposited solid was filtered, washed with water, dried, and recrystallized from ethanol to acquire yellow crystals, mp. >300 °C, Yield (75%), Anal. Calcd. for C_25_H_14_Cl_2_N_4_O (457.3):C, 65.66; H, 3.09; N,12.25. Found: C, 65.68; H, 3.11; N, 12.22%. FTIR (ν, cm^− 1^): 3377, 3182 (NH_2_), 2201 (CN), 1710 (C = O), 1631 (C = N). ^1^H NMR (DMSO-*d*_6_, *δ*, ppm): 9.05 (d, 1H, C5-H), 8.91 (s, 1H, C4-H quinoline), 8.70–7.61 (m, 10 H, Ar-H + CH pyridine), 7.07 (s, 2 H, NH_2_, exchangeable).The mass(*m/z%*): 457.13(40.75), 404.78(100), 337.99(57.35), 269.70(53.91), 238.76(47.46), 184.33(43.99).

### 4-(3-chlorobenzo[f]quinolin-2-yl)-6-(4-chlorophenyl)pyridin-2(1 H)-one (10)

A mixture of chalcone **3** (0.01 mol, 3.78 g), ethyl cyanoacetate (0.01 mol, 1.13 mL), and ammonium acetate (0.03 mol, 2.31 g) was fused for 10 h. The solid obtained was filtered and then recrystallized from ethanol to produce pale-yellow crystals, mp. 170–172 °C, Yield (85%), Anal. Calcd. for C_24_H_14_Cl_2_N_2_O (417.3):C, 69.08; H, 3.38; N,6.71. Found: C, 69.11; H, 3.35; N, 6.74%. FTIR (ν, cm^− 1^): 3201 (NH), 1668 (C = O). ^1^H NMR (DMSO-*d*_6_, *δ*, ppm): 9.07 (s, 1H, NH, exchangeable), 8.99(d, 1H, C5-H), 8.78 (s, 1H, C4-H quinoline), 8.56–7.30 (m, 11 H, Ar-H).The mass(*m/z%*): 417.02(32.11), 389.79(57.84), 341.32(34.94), 264.10(89.46), 196.30(100), 147.30(76.59), 52.35(84.90).

### Insecticidal activity

#### Insect colony

The field strain of *Culex pipiens* egg rafts was collected from Abu Rawash, Giza, Egypt, while the Lab strain egg rafts were provided by the Research and Training Center, Faculty of Science, Ain Shams University. The eggs were hatched in glass pots containing distilled water. The larvae were fed on crushed fish food and incubated in controlled conditions at 27–29 °C, 70–80% R.H., and 12-h photoperiod^47,48^, to obtain larvae in the early third instar of development.

#### Larvicidal bioassays

The larvicidal activity of chalcone derivatives was assessed according to^49^. The chalcone derivatives were dissolved in DMSO to prepare the stock solutions of the tested compounds. Seven dilutions (100, 150, 200, 250, 300, 350 and 400 µg/mL) for the Lab strain and six concentrations (150, 200, 250, 300, 350 and 400 µg/mL) for the field strain were prepared in distilled water. Temphose was used as a reference larvicide for mosquitoes with concentrations viz., 25, 50, 75, 100, 125, and 150 µg/mL. 60 mL of each concentration was added to plastic cups (100 mL), then 20 *C. pipiens* larvae. An equivalent volume of DMSO was added to distilled water as a negative control. Both treatment and control were replicated 3 times. Larval mortality was determined after 48 h of exposure by counting the number of dead larvae relative to live^19^.

### Statistical analysis

The LC values, slope, chi-square, and probability of *C. pipiens* larvae lab and field strains were estimated by LDP-line according to probit analysis^50^. Resistance ratios of the *C. pipiens* field strain were calculated and classified as low resistance ratio (RR < 5), moderate (5 < RR < 10), and high (RR > 10) according to^51^.

### Ethics declaration

This study was approved by the Research Ethics Committee at Ain Shams University (Approval code: ASU-SCI/CHEM/2024/5/1) and was performed by the guidelines of the National Institute of Health (NIH). All methods are reported by the ARRIVE guidelines.

## Supplementary Information

Below is the link to the electronic supplementary material.

Supplementary Material 1

## Data Availability

All data generated or analyzed during this study are included in this published article and its supplementary information file.
