# Supplementary material for: Synthesis and insecticidal activity of some pyrazole, pyridine, and pyrimidine candidates against Culex pipiens L. larvae
Source: Sci Rep. 2025 Nov 29;15:42919. doi: 10.1038/s41598-025-27807-y (PMC12672691; doi:10.1038/s41598-025-27807-y)
Supplement: Supplementary file 1 — Supplementary Material 1 [file 41598_2025_27807_MOESM1_ESM.docx]

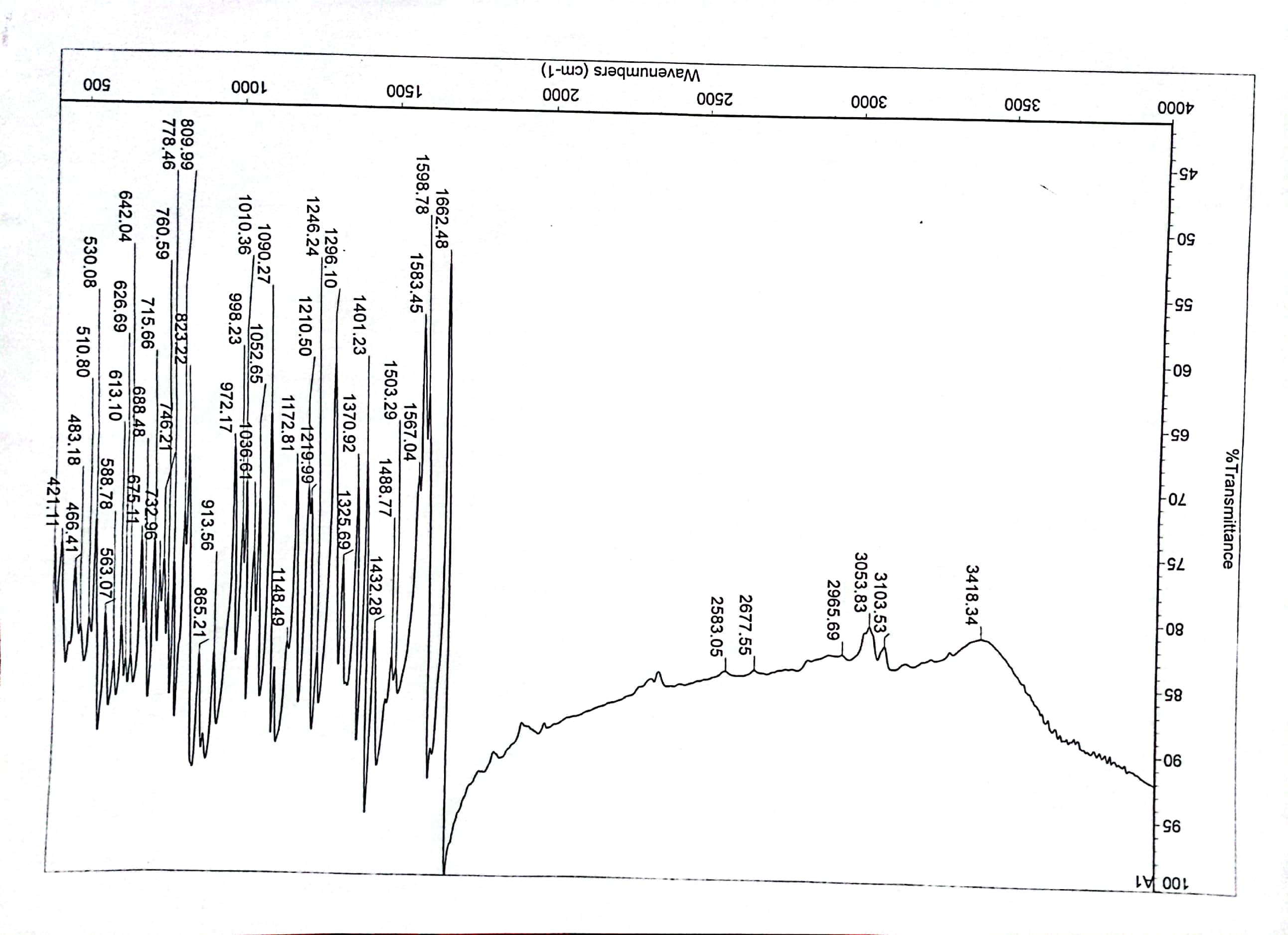


**Figure 1**:IR spectrum of compound **3**


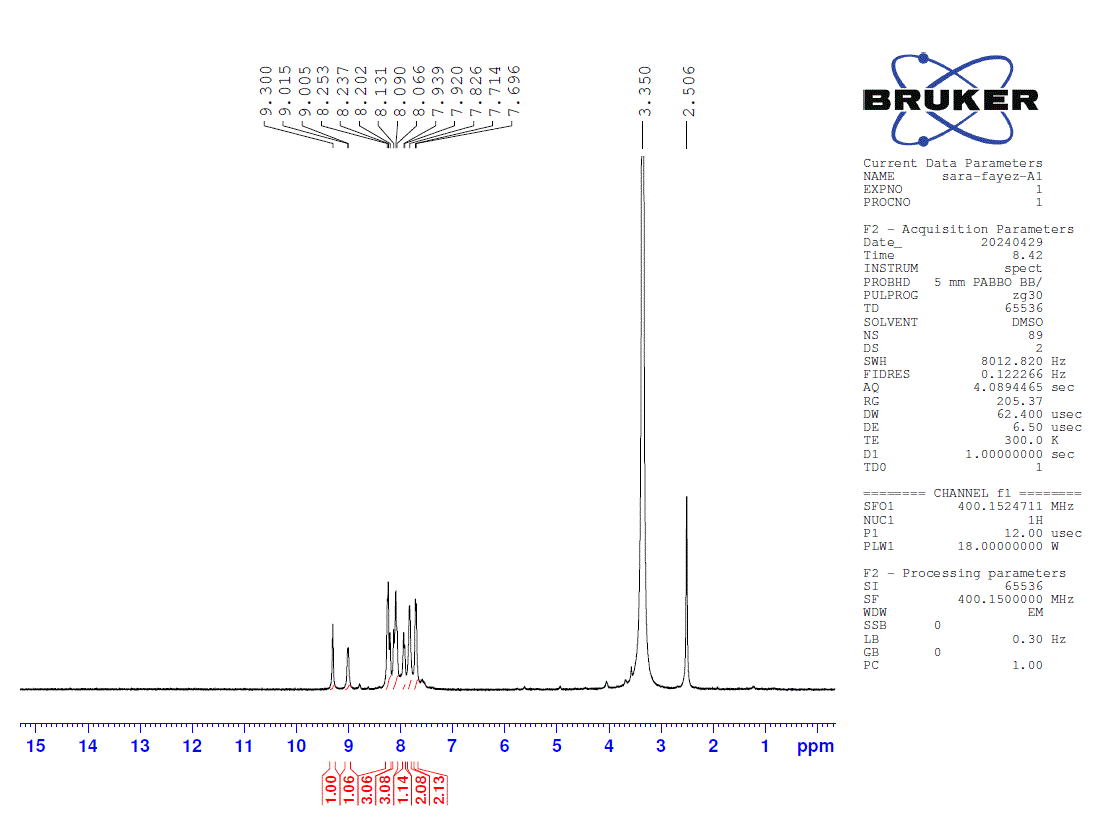


**Figure 2**:^1^H-NMR (DMSO-d6) spectrum of compound **3**


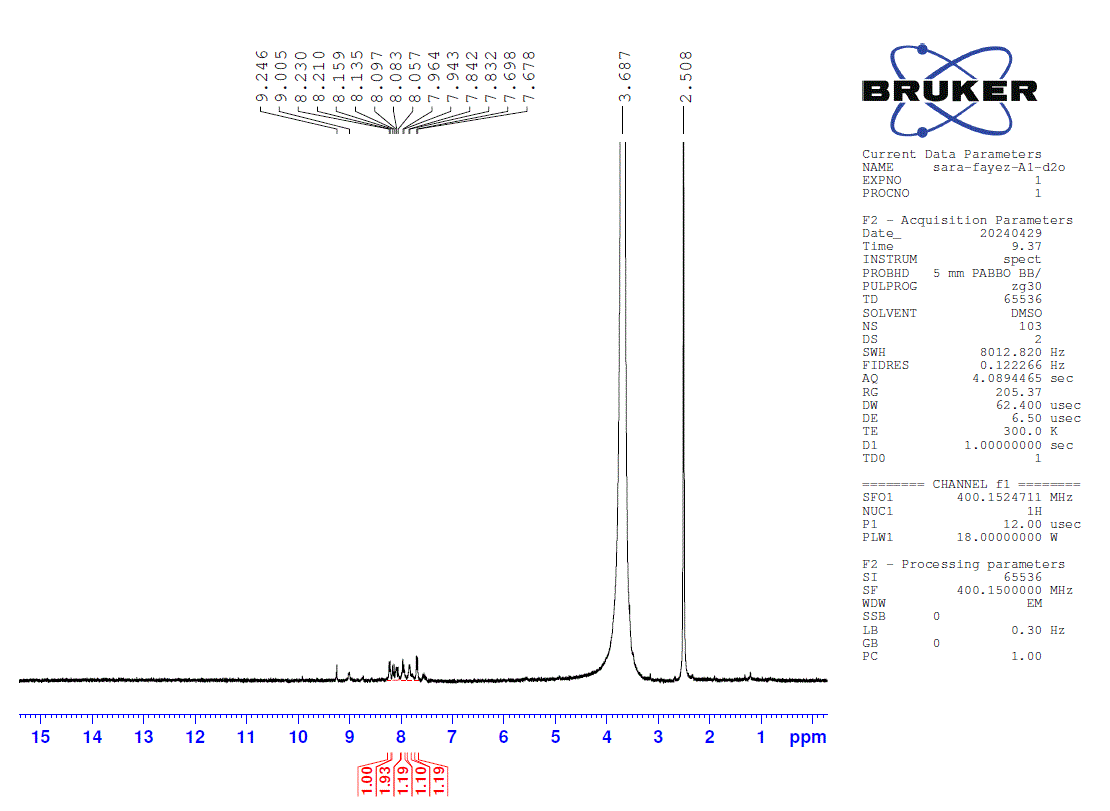


**Figure 3**:^1^H-NMR (DMSO- *d*_6_ +D_2_O) spectrum of compound **3**

**
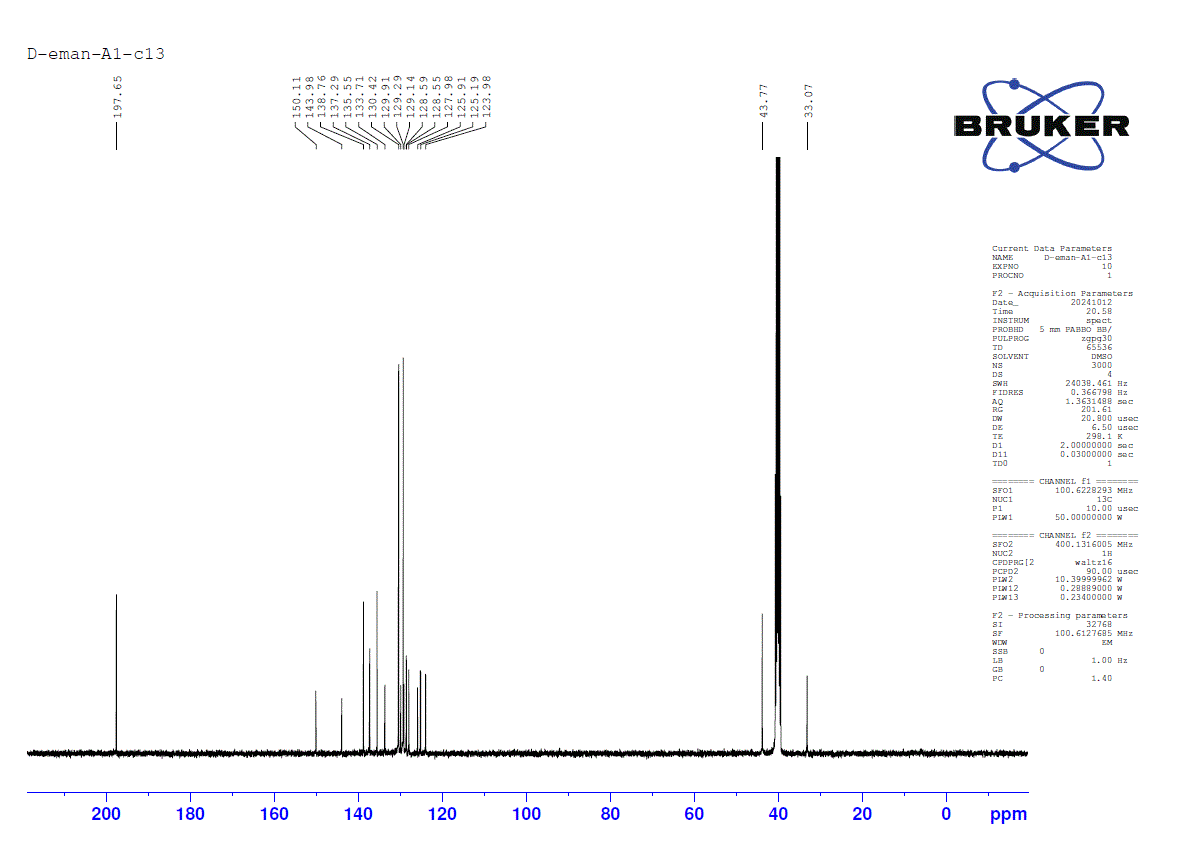
**

**Figure 4**:^13^C-NMR spectrum of compound **3**

**
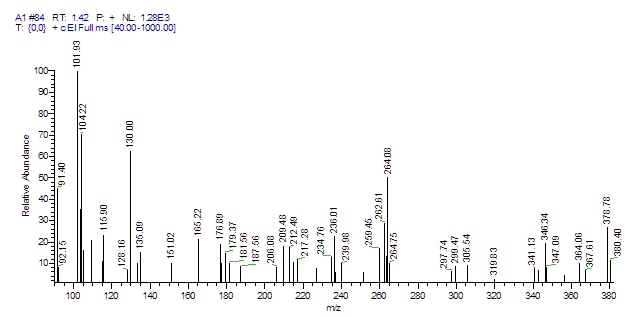
**

**Figure 5:** Mass spectrum of compound **3**


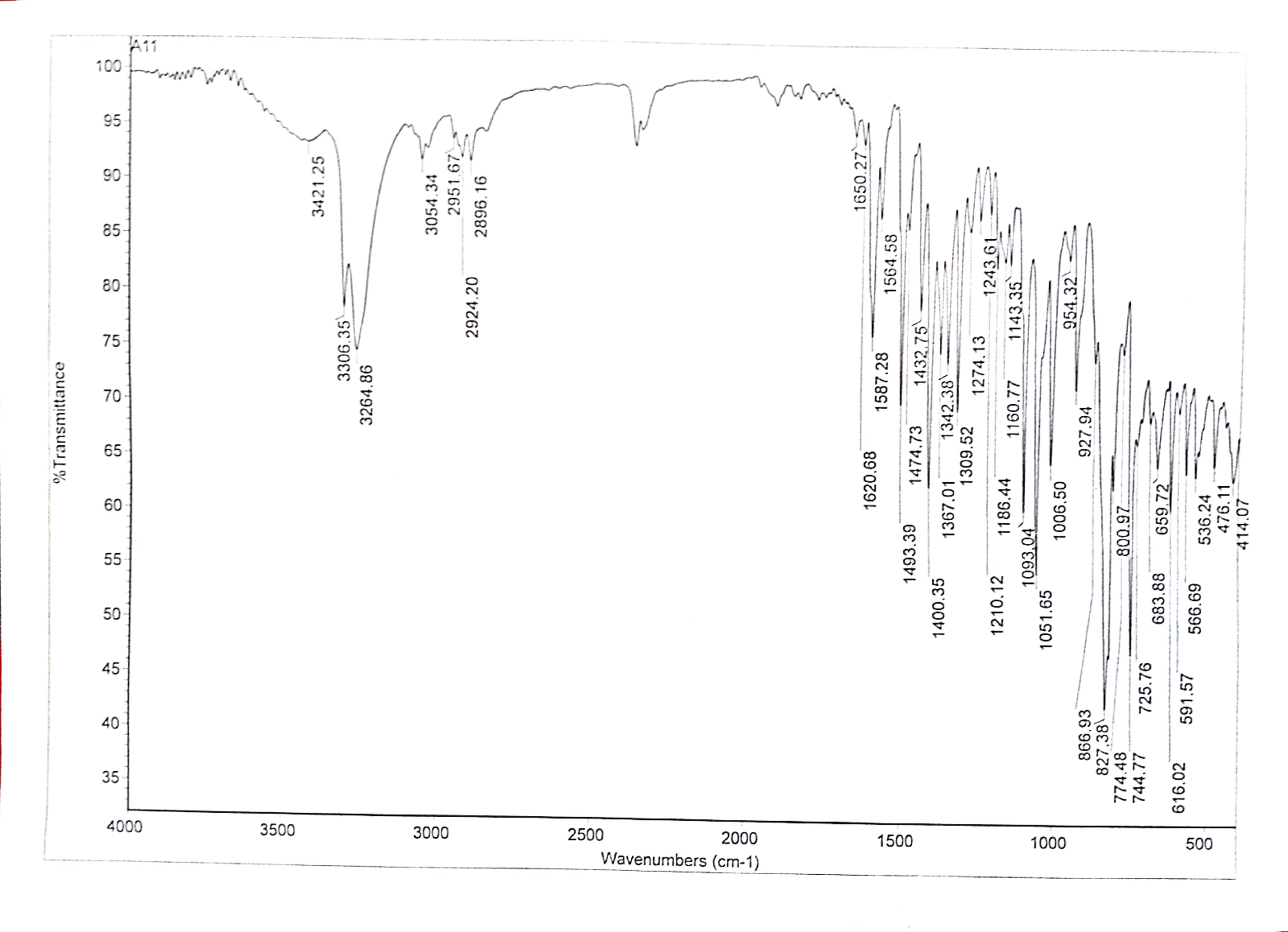


**Figure 6**:IR spectrum of compound **4**


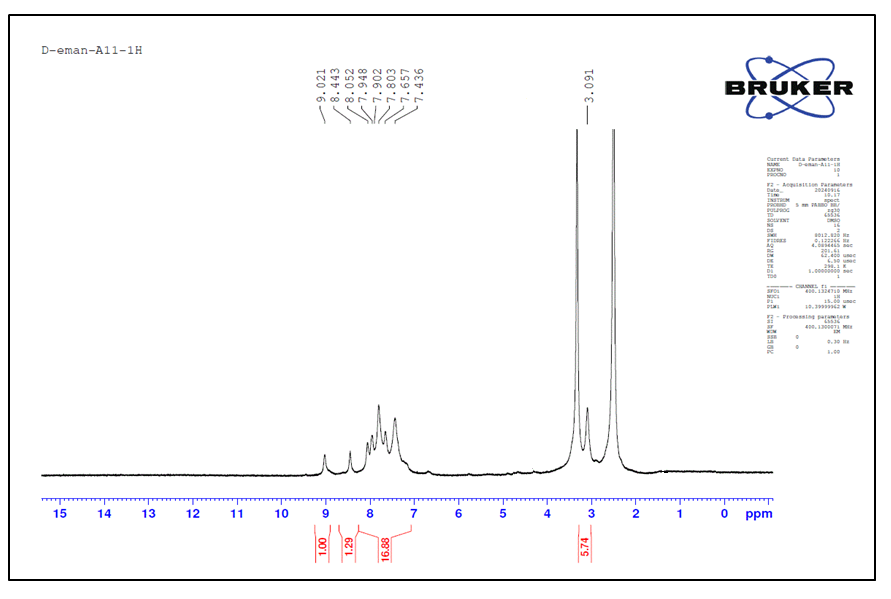


**Figure 7**:^1^H-NMR (DMSO- *d*_6_) spectrum of compound **4**

**
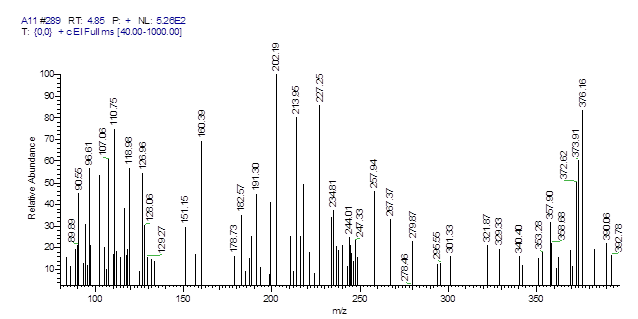
**

**Figure 8**: Mass spectrum of compound **4**


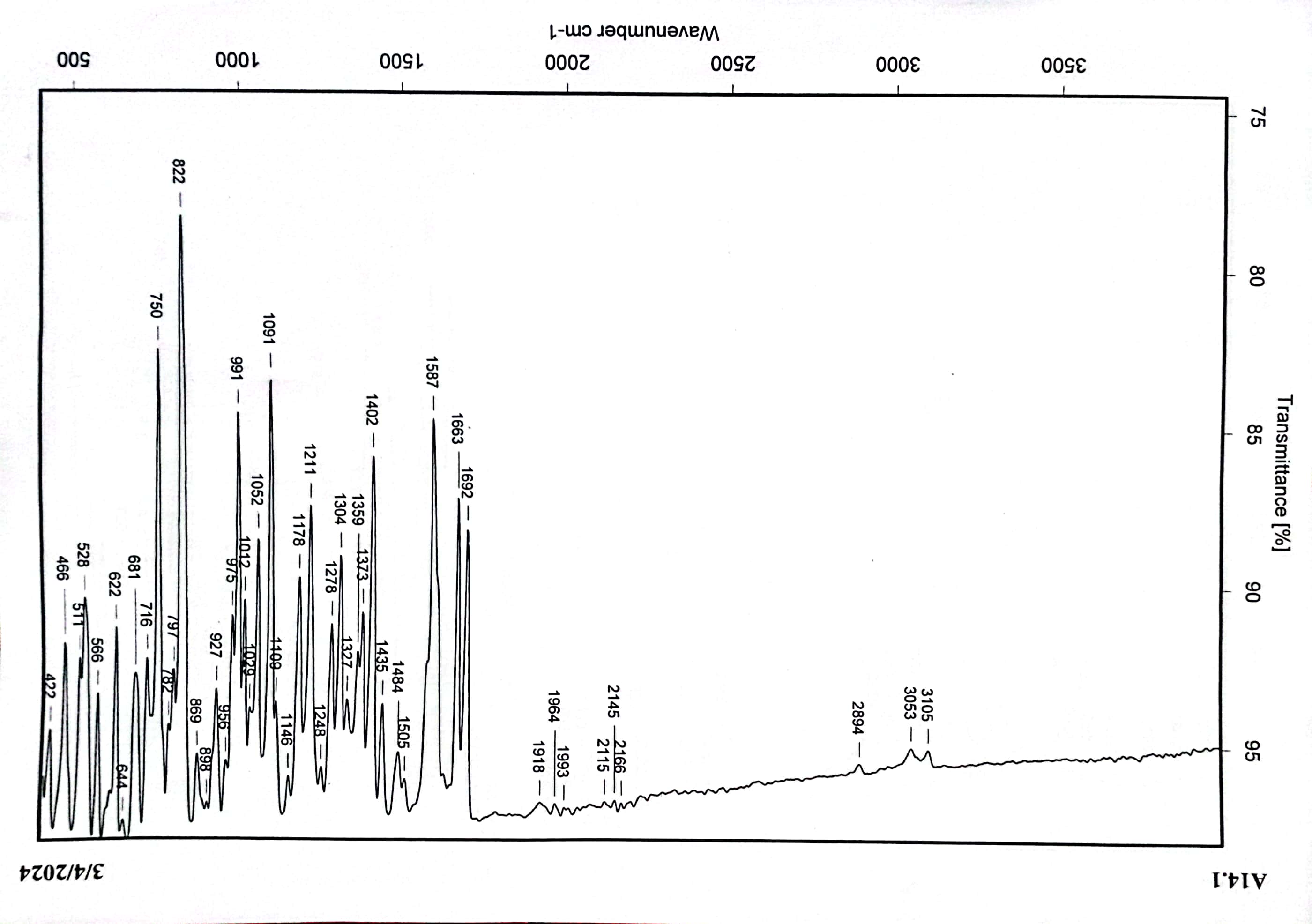


**Figure 9**:IR spectrum of compound **5**


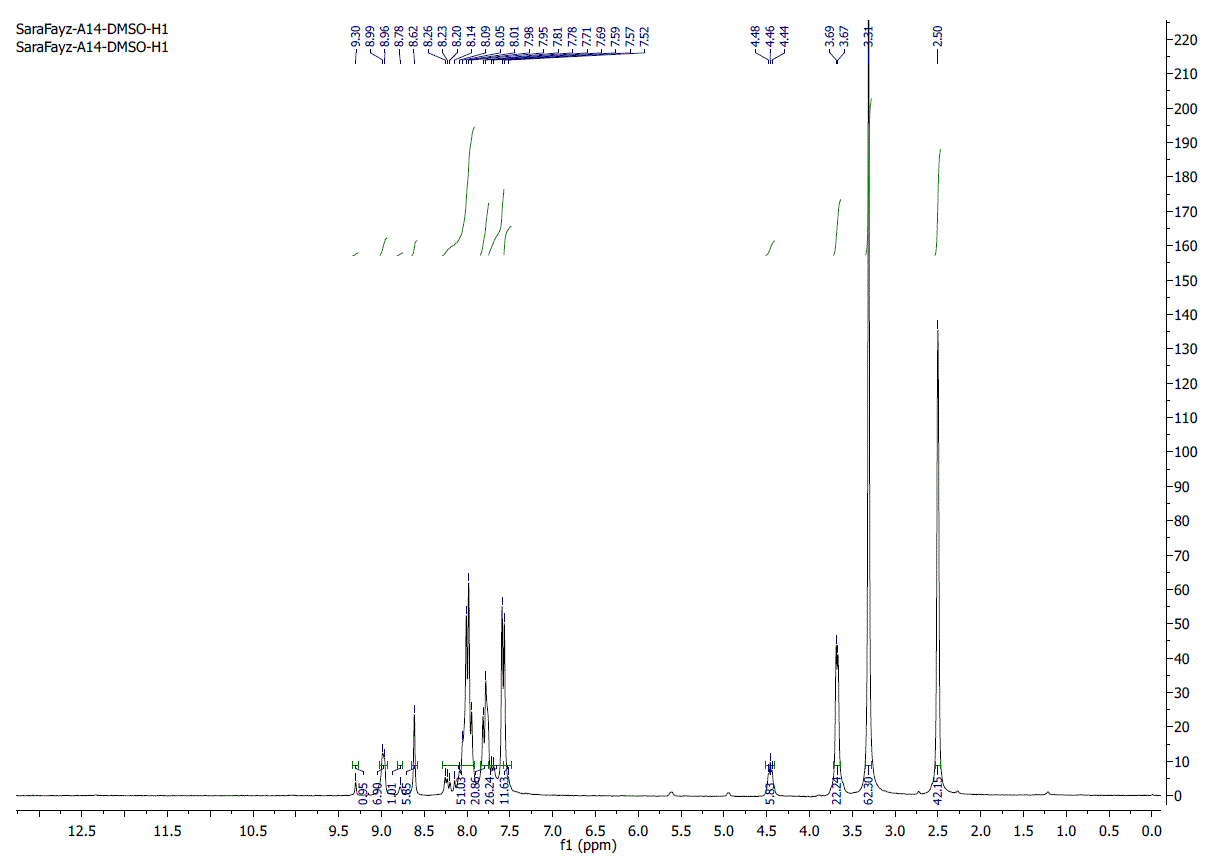


**Figure 10:**^1^H-NMR (DMSO- *d*_6_) spectrum of compound **5**


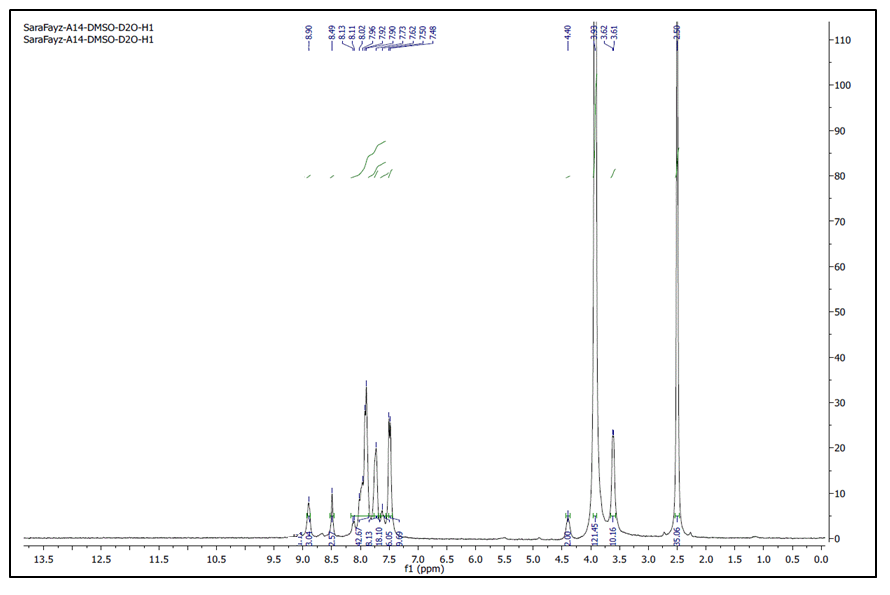


**Figure 11:**^1^H-NMR (DMSO- *d*_6_ +D_2_O) spectrum of compound **5**


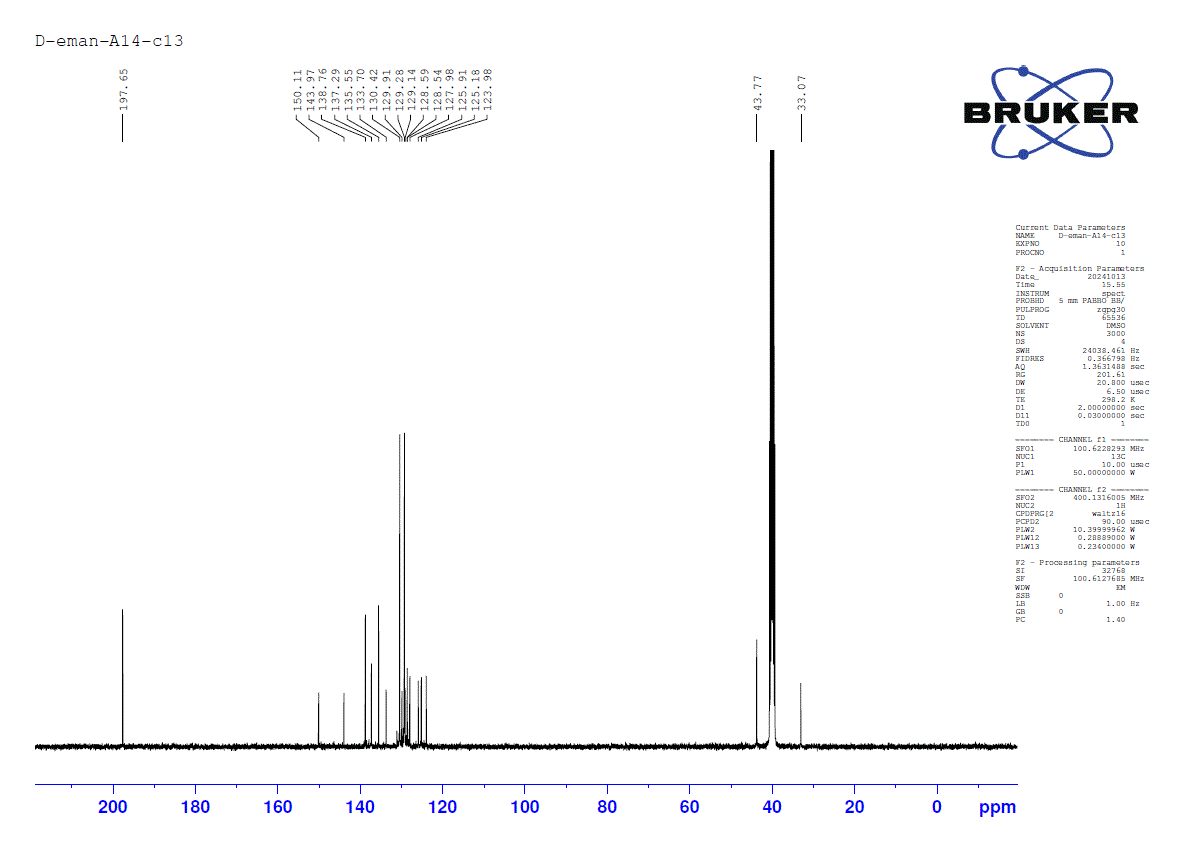


**Figure 12:**^13^ C-NMR spectrum of compound **5**


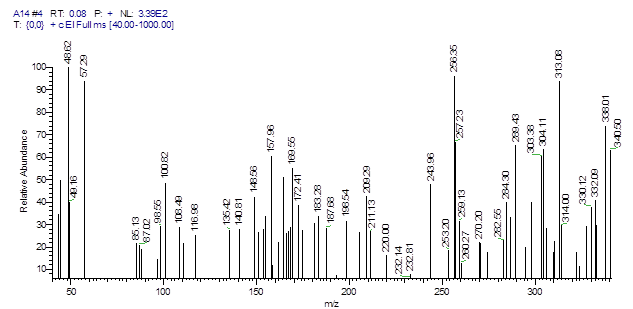


**Figure 13:** Mass spectrum of compound **5**


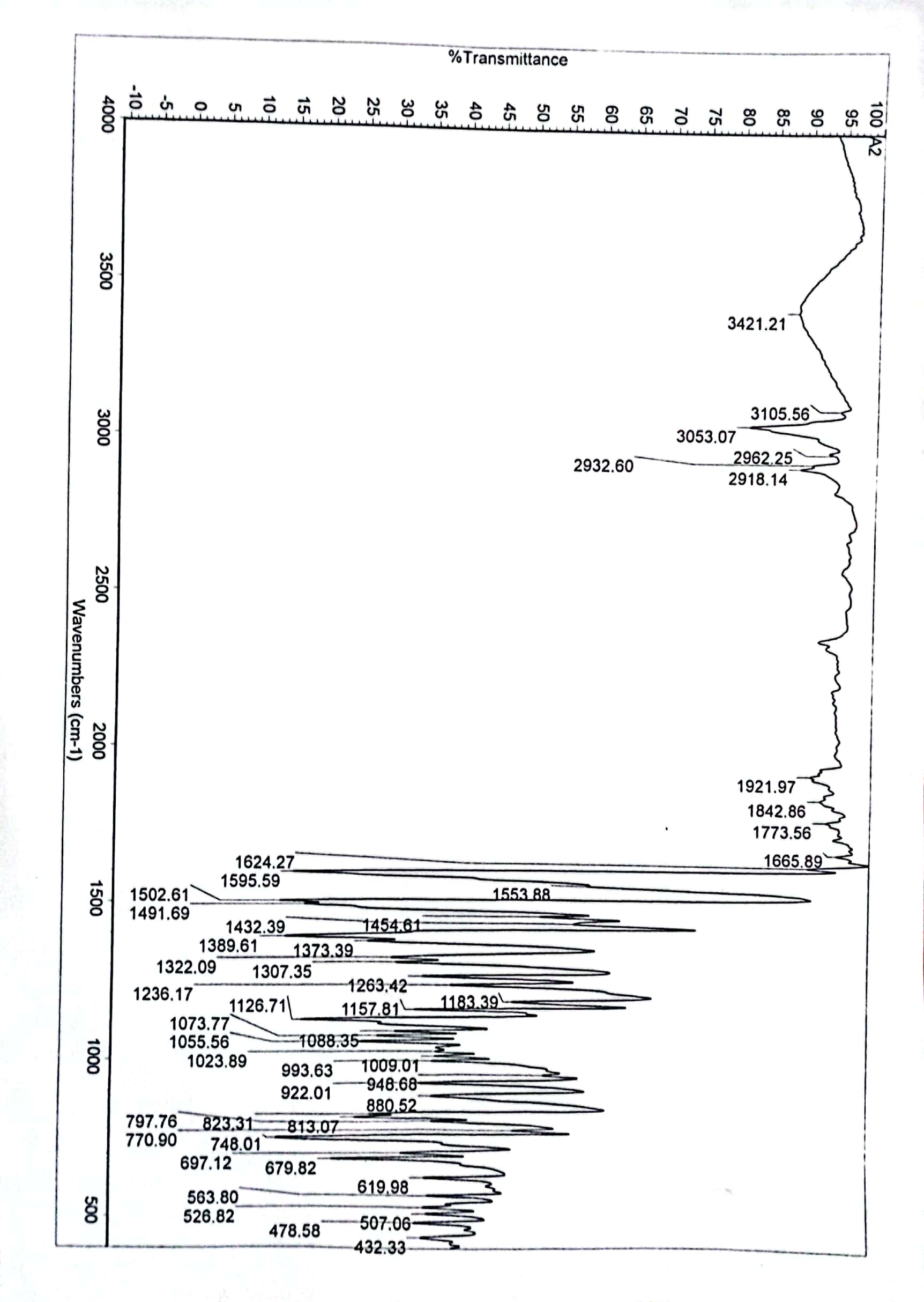


**Figure 14:** IR spectrum of compound **6**


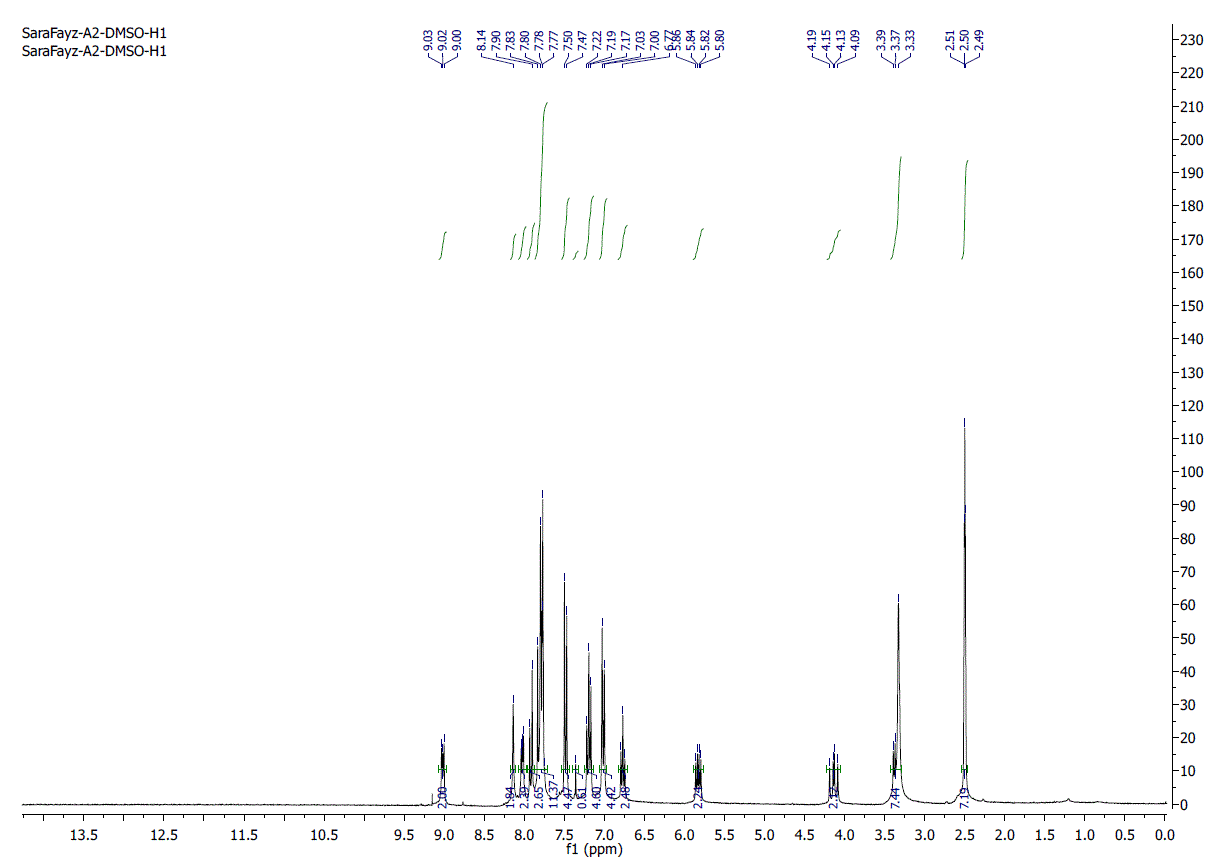


**Figure 15:**^1^H-NMR (DMSO- *d*_6_) spectrum of compound **6**


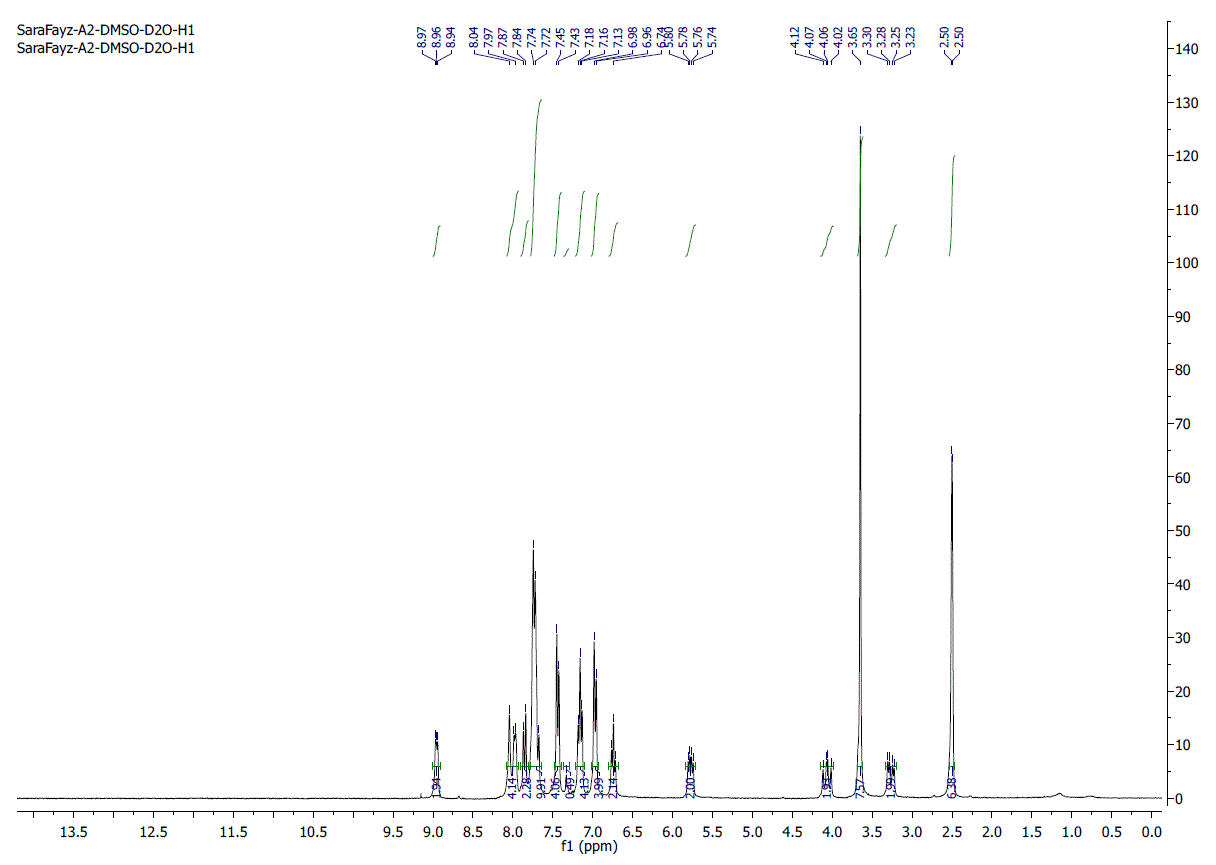


**Figure 16:**^1^H-NMR (DMSO- *d*_6_ +D_2_O) spectrum of compound **6**

**
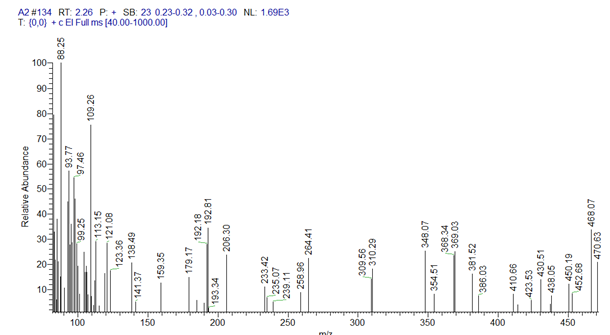
**

**Figure 17:** Mass spectrum of compound **6**


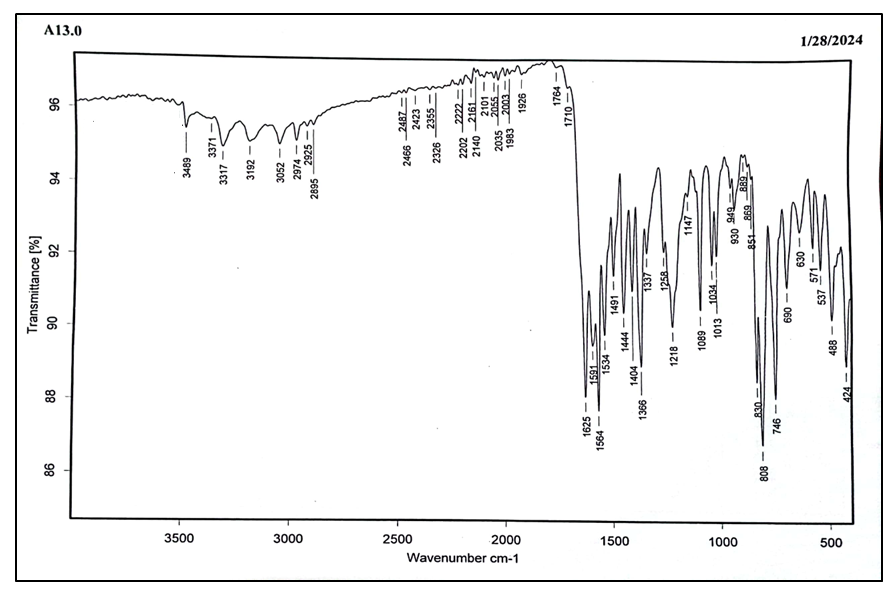


**Figure 18**:IR spectrum of compound **7**


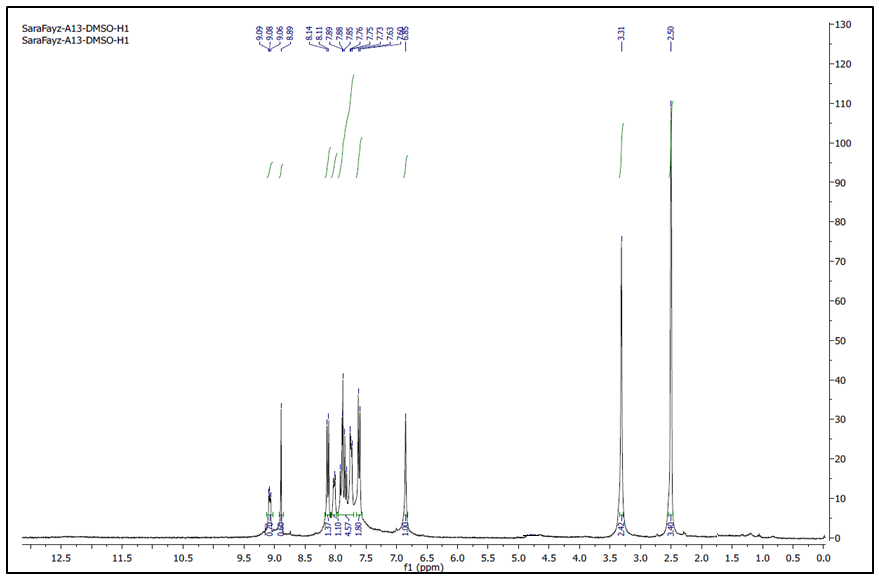


**Figure 19:**^1^H-NMR (DMSO- *d*_6_) spectrum of compound **7**


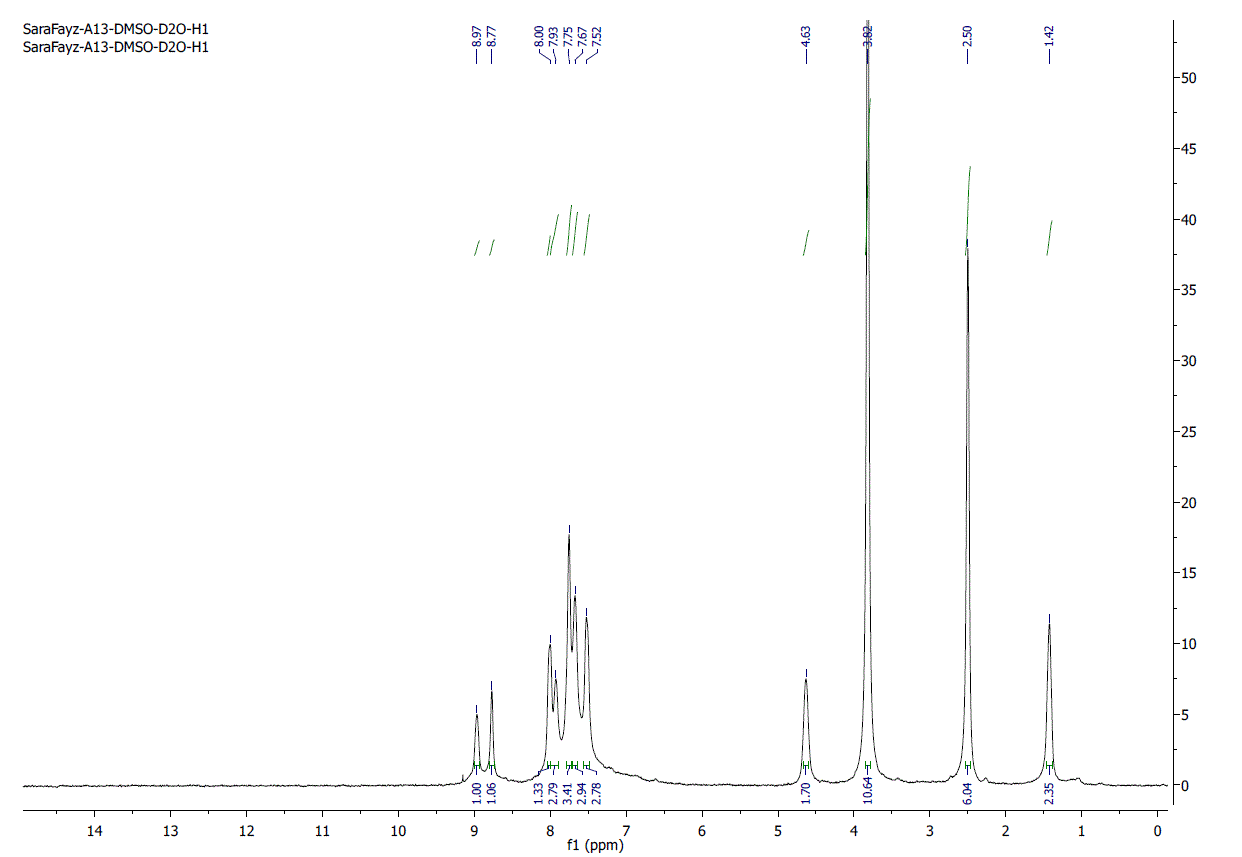


**Figure 20**:^1^H-NMR (DMSO- *d*_6_ +D_2_O) spectrum of compound **7**


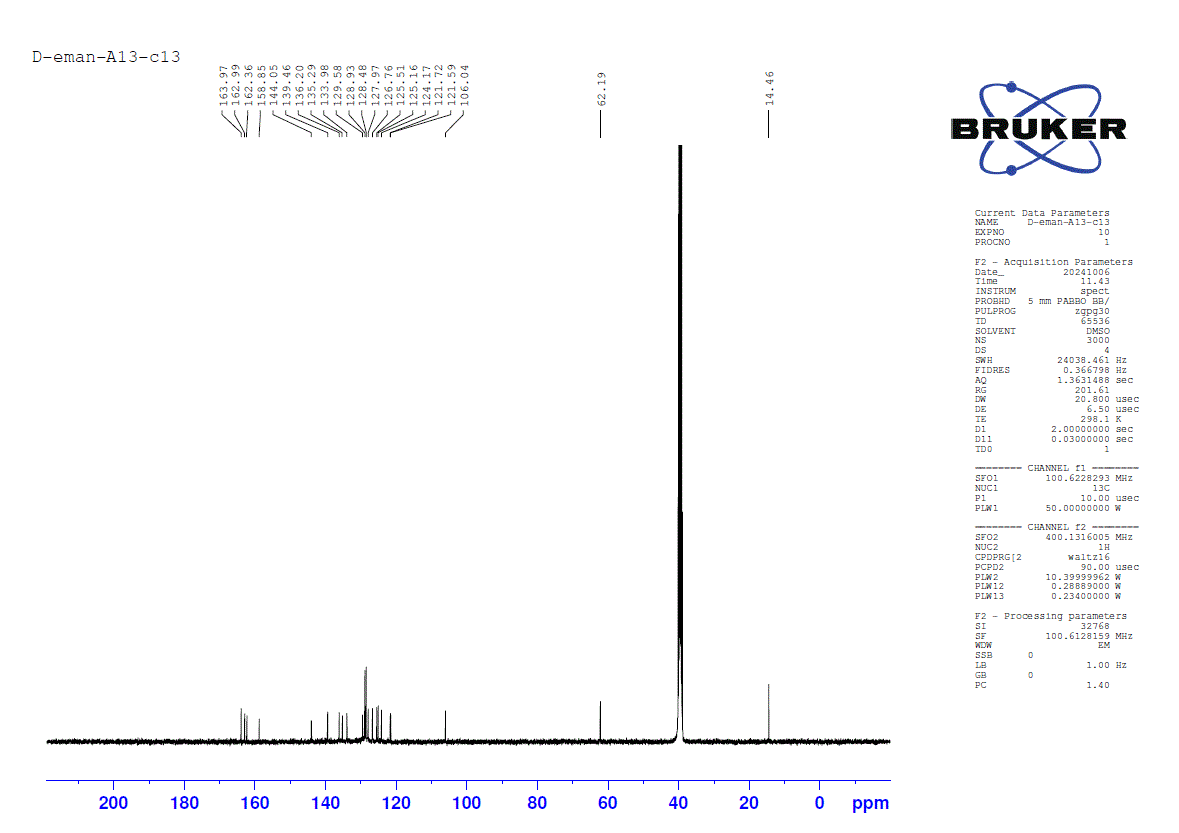


**Figure 21**:^13^ C-NMR spectrum of compound **7**

**
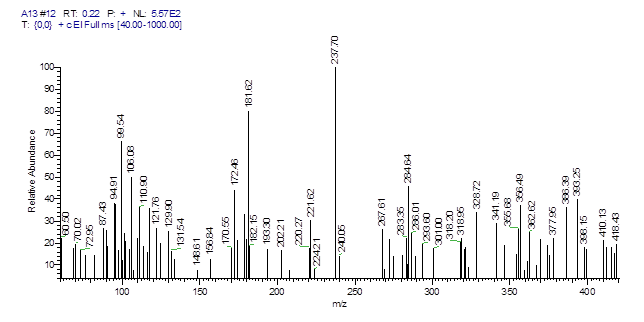
**

**Figure 22:** Mass spectrum of compound **7**


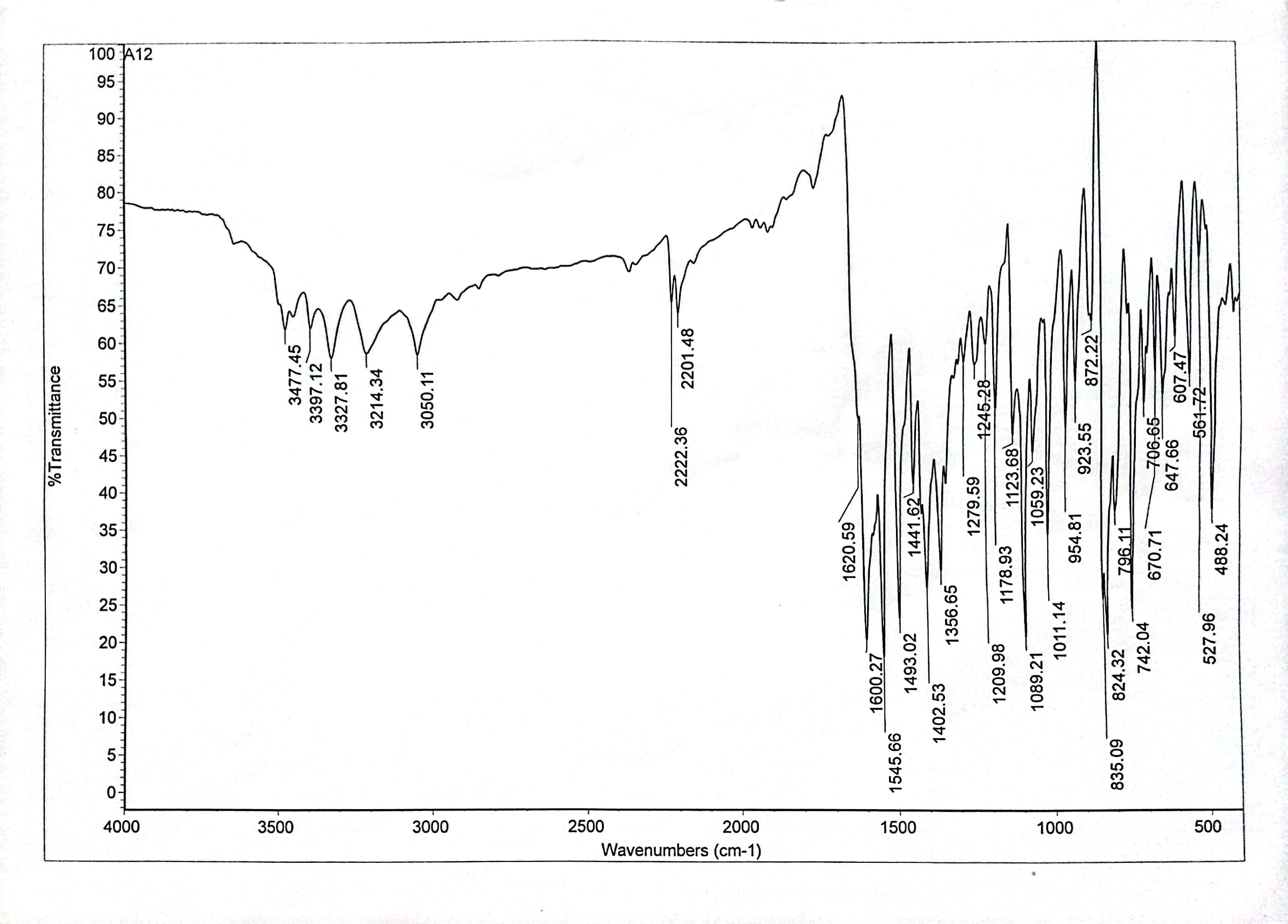


**Figure 23:** IR spectrum of compound **8**


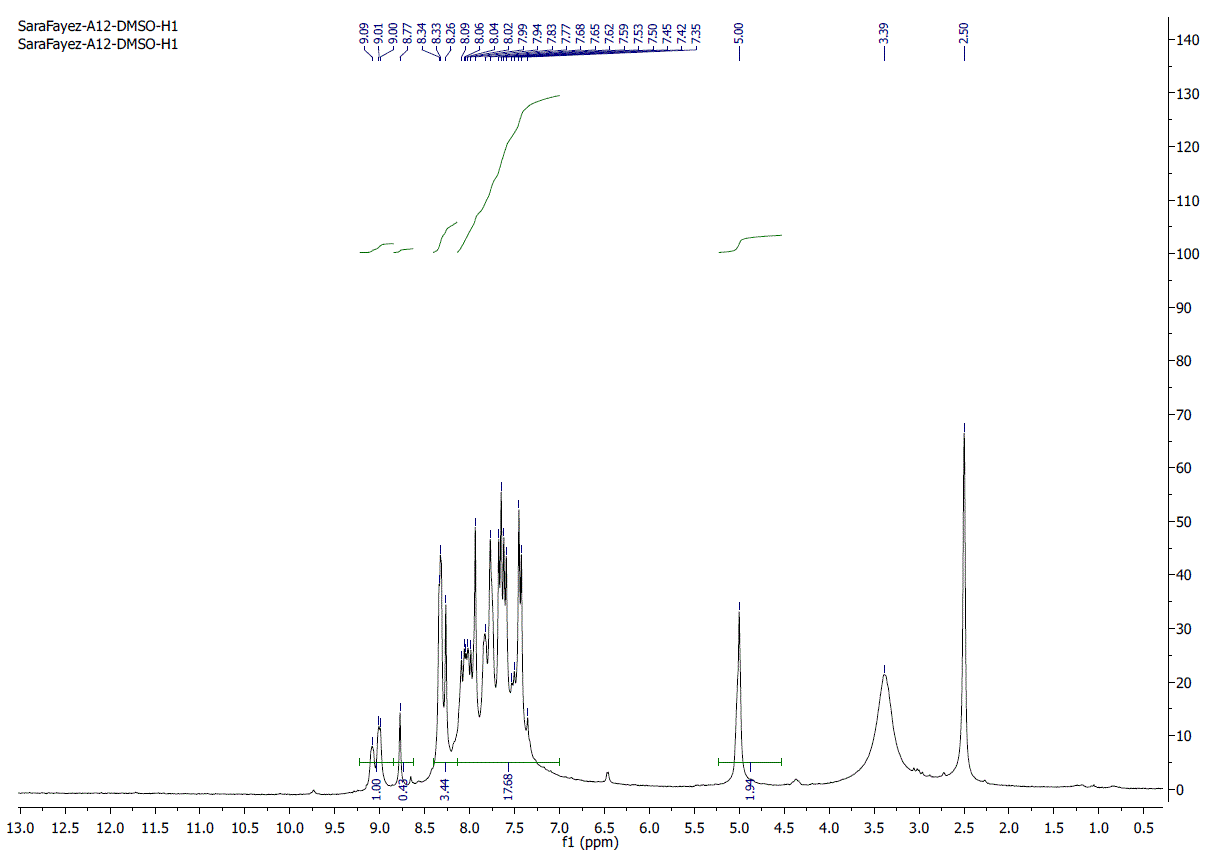


**Figure 24:**^1^H-NMR (DMSO- *d*_6_) spectrum of compound **8**

**
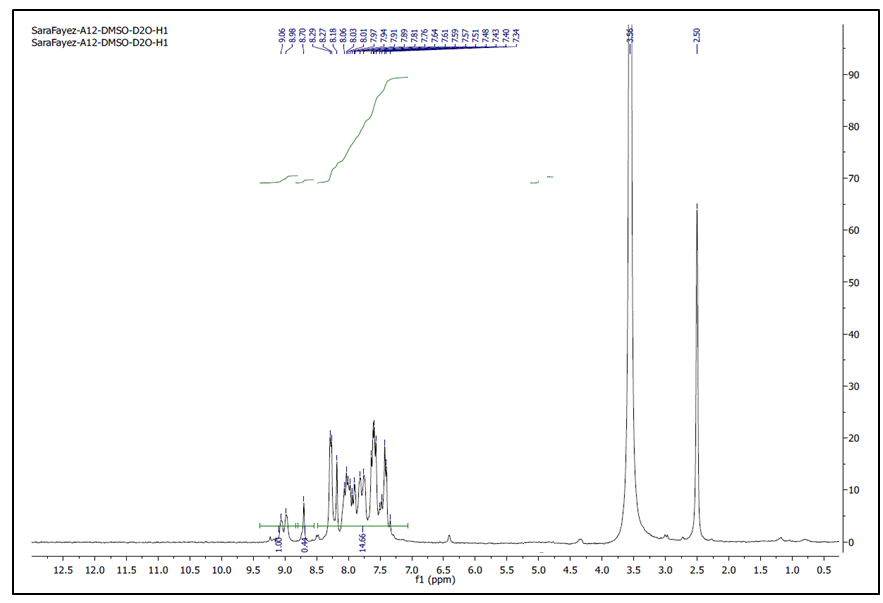
**

**Figure 25:**^1^H-NMR (DMSO- *d*_6_ +D_2_O) spectrum of compound **8**

**
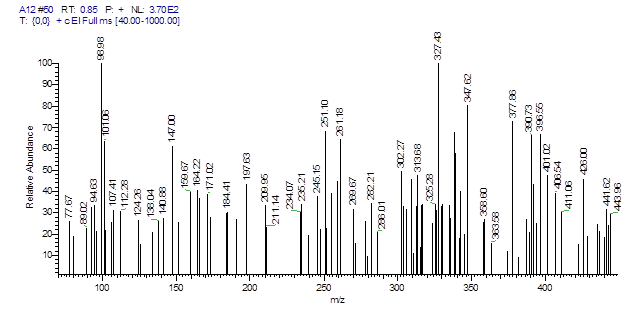
**

**Figure 26:** Mass spectrum of compound **8**


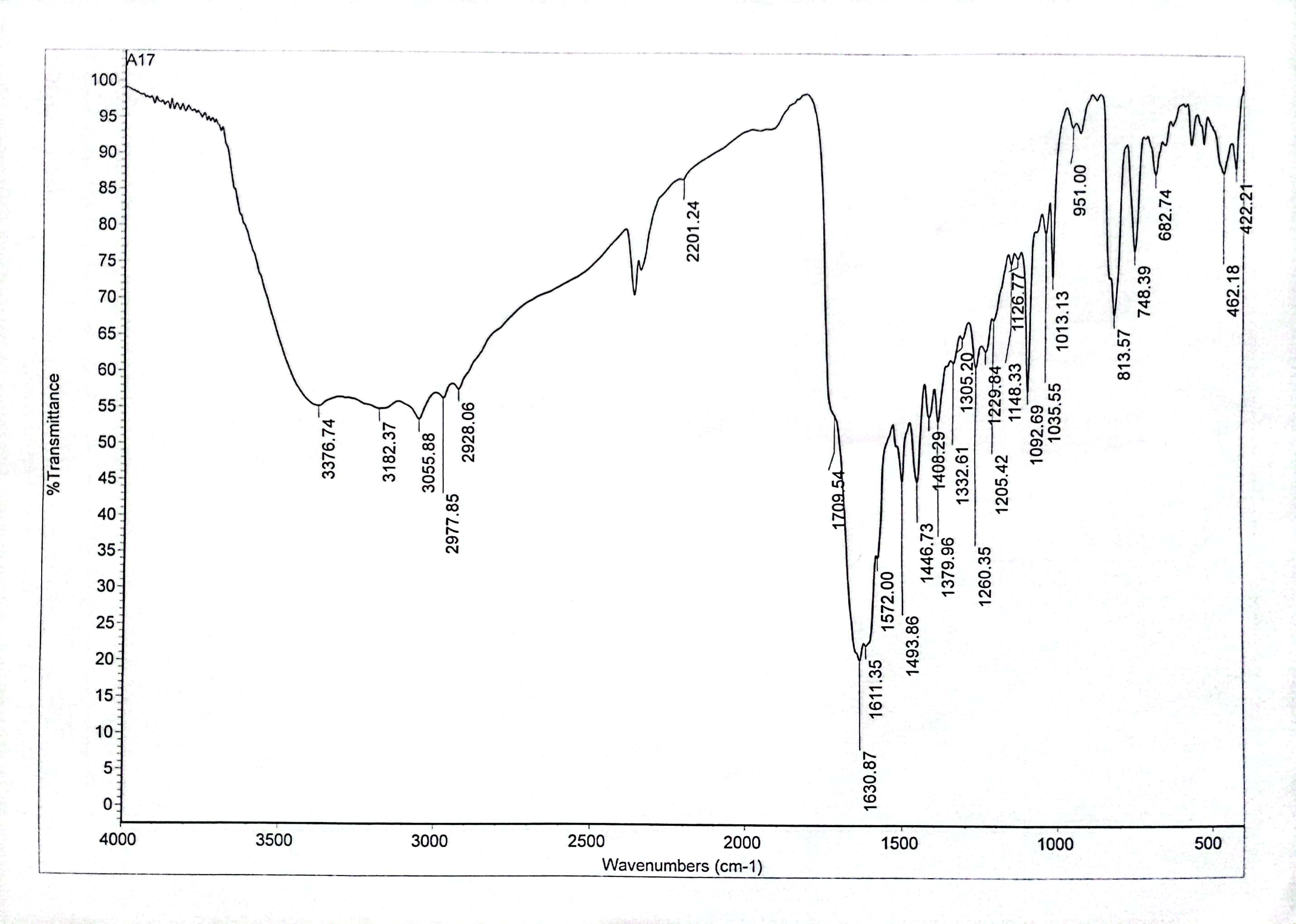


**Figure 27:** IR spectrum of compound **9**

**
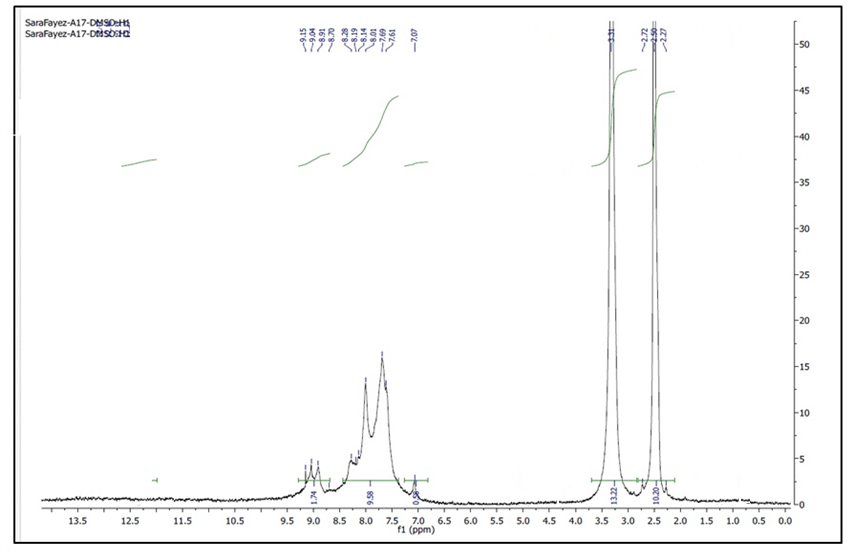
**

**Figure 28:**^1^H-NMR (DMSO- *d*_6_) spectrum of compound **9**

**
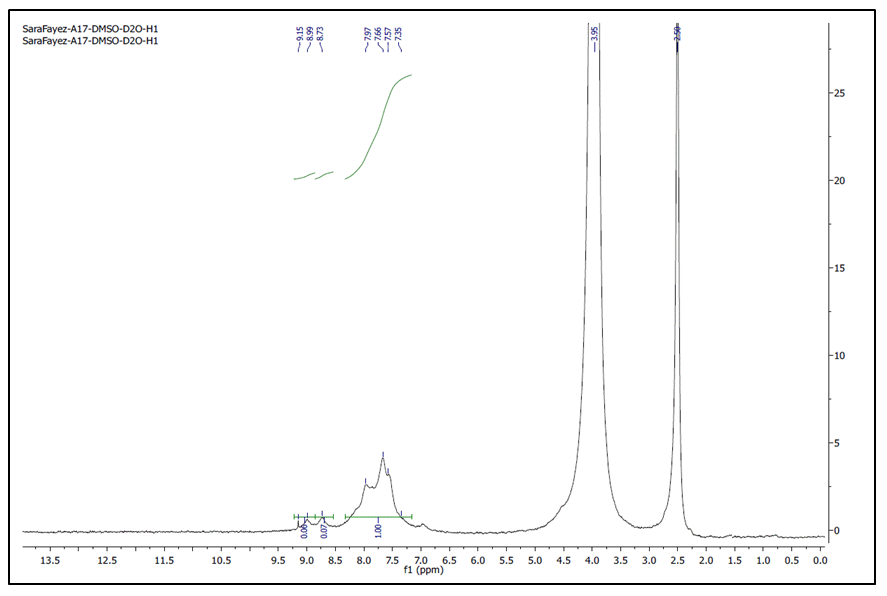
**

**Figure 29:**^1^H-NMR (DMSO- *d*_6_ +D_2_O) spectrum of compound **9**

**
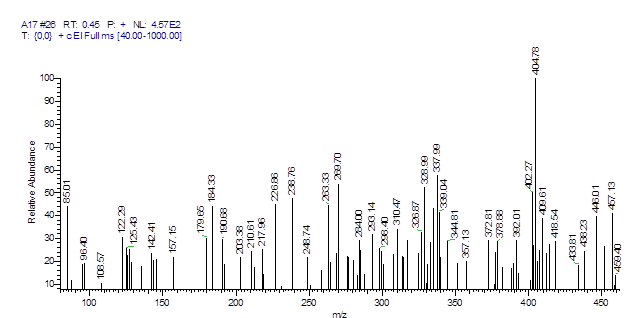
**

**Figure 30**: Mass spectrum of compound **9**


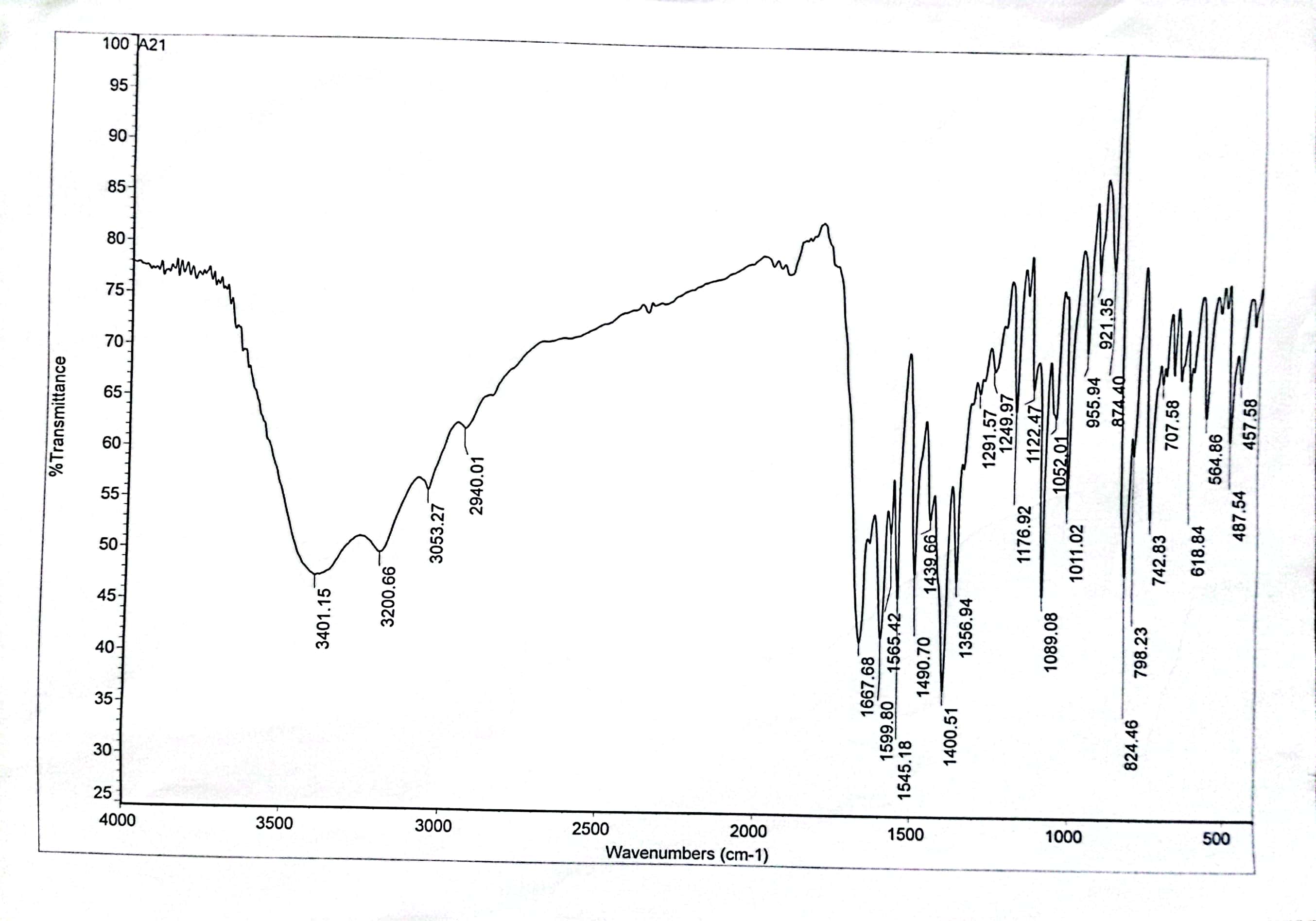


**Figure 31:**IR spectrum of compound **10**

**
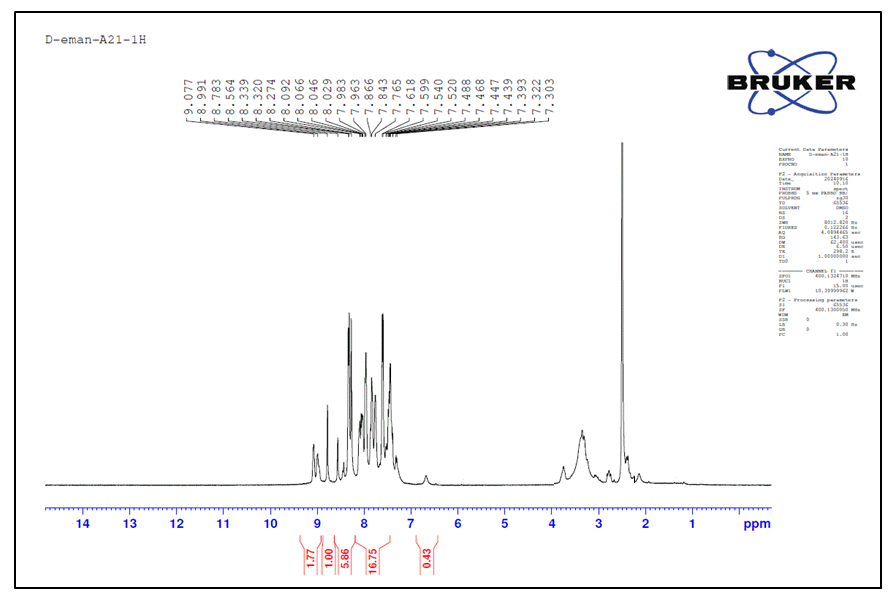
**

**Figure 32:**^1^H-NMR (DMSO- *d*_6_) spectrum of compound **10**


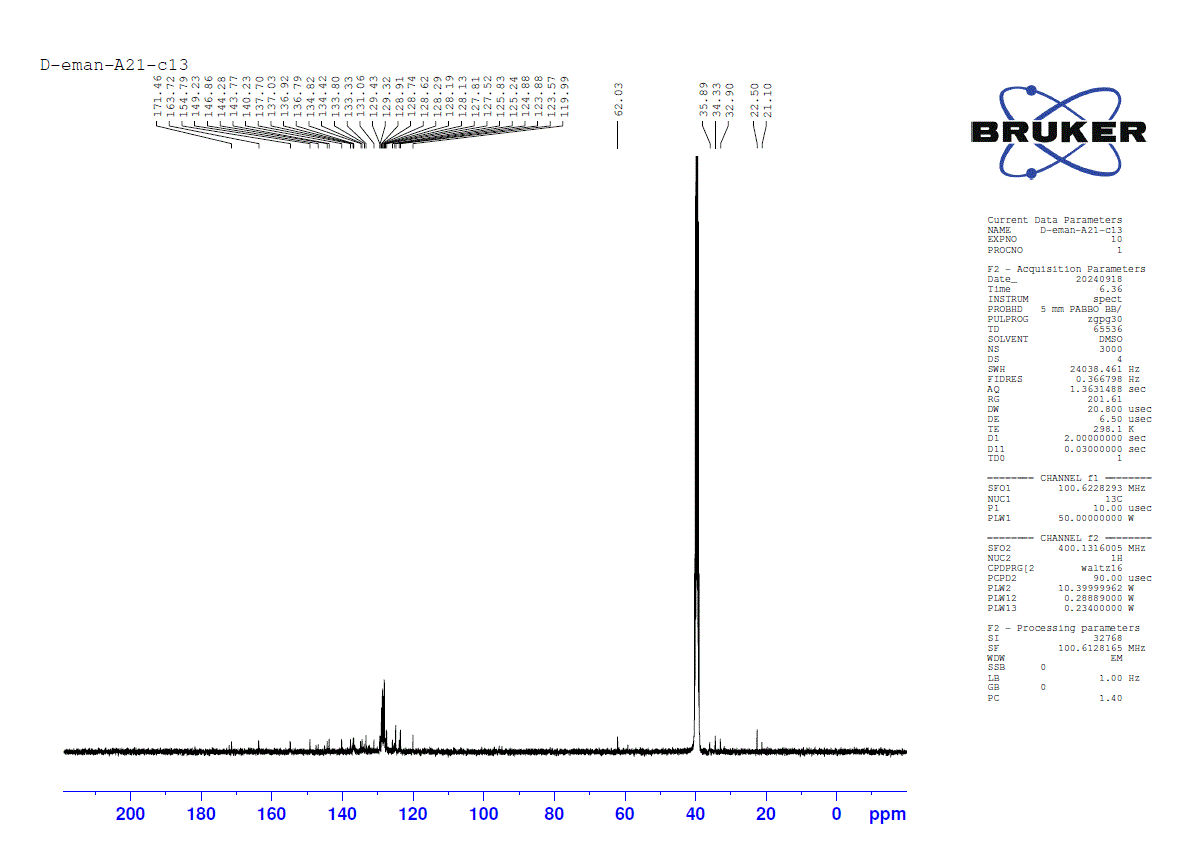


**Figure 33:**^13^C-NMR spectrum of compound **10**

**
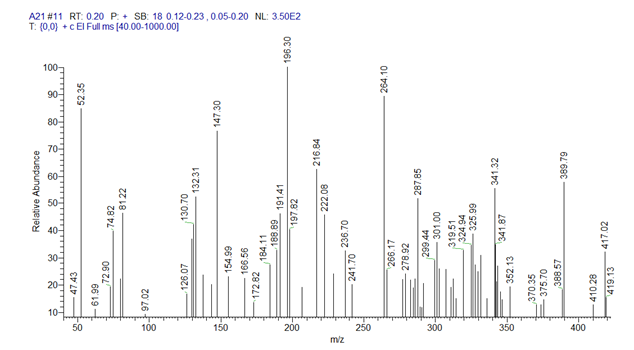
**

**Figure 34:** Mass spectrum of compound 10
